# New sulfonamide derivatives as HDAC inhibitors and apoptosis inducers: design, synthesis, *in silico* study and anticancer evaluation

**DOI:** 10.1039/d6ra05385h

**Published:** 2026-10-27

**Authors:** Omkulthom Al kamaly, Esmail M. El-Fakharany, Yousra A. El-Maradny, Lama A. Alshabani, Aisha Alsfouk, Abdallah E. Abdallah

**Affiliations:** a Department of Pharmaceutical Sciences, College of Pharmacy, Princess Nourah bint Abdulrahman University P.O. Box 84428 Riyadh 11671 Saudi Arabia; b Protein Research Department, Genetic Engineering and Biotechnology Research Institute (GEBRI), City of Scientific Research and Technological Applications (SRTA-City) New Borg Al-Arab City 21934 Alexandria Egypt; c Pharmaceutical Medicinal Chemistry & Drug Design Department, Faculty of Pharmacy (Boys), Al-Azhar University Cairo 11884 Egypt abdulla_emara@azhar.edu.eg

## Abstract

Cancer statistics indicate that more research is required to combat such a global fatal disease. In an attempt to develop an effective anticancer scaffold, we designed and synthesized new sulfonamide derivatives for potential histone deacetylase (HDAC) inhibition. The new derivatives showed antiproliferative activity against three cancer cell lines: HepG-2 (hepatoma), A549 (lung carcinoma), and MCF-7 (breast carcinoma). The most sensitive cancer cell line to the new derivatives was MCF-7, followed by A549 then HepG-2. Compound 10 emerged as the most effective candidate, especially against MCF-7, where it showed an IC_50_ of 13.56 ± 1.36 µM. Meanwhile, it had an IC_50_ of 20.03 ± 2.17 and 34.71 ± 4.62 µM against A549 and HepG-2 cells, respectively. Interestingly, molecule 10 exhibited a markedly higher *in vitro* selectivity toward the tested cancer cell lines than the reference drug 5-fluorouracil (5-FU), with selectivity indices of 8.74 ± 0.88, 5.92 ± 0.74, and 3.41 ± 0.39 against MCF-7, A549, and HepG-2 cells, respectively, compared with 1.59 ± 0.13, 1.16 ± 0.09, and 1.67 ± 0.14 for 5-FU, respectively. These findings indicate greater *in vitro* selectivity of compound 10 toward the tested cancer cells relative to the normal WI-38 cell line; however, they should not be interpreted as evidence of superior overall safety compared with 5-FU. Furthermore, 10 caused inhibition to HDAC especially HDAC-2, showing an IC_50_ of 2.27 ± 0.07 µM, in comparison to the reference molecule entinostat (IC_50_ of 1.21 ± 0.04 µM). The Annexin V-FITC/PI assay indicated that apoptosis was the predominant mode of cell death caused by compound 10. Compound 10 caused an approximately 15-fold increase in the total number of apoptotic MCF-7 cells relative to untreated control cells. Meanwhile, compound 10 increased the expression levels of apoptotic markers p53, and the protein levels of caspase-9 in MCF-7 cells. Whereas it decreased the expression levels of Bcl-2 and VEGF. The docking data that revealed insights into the binding pattern of compound 10 to HDAC-2, were consistent with the biological data. Finally, this study suggests that compound 10 may serve as a lead molecule for further optimization toward new anticancer drugs.

## Abstract 2

Compound 10 inhibited HDAC2 with correct binding mode, induced apoptosis and elevated apoptosis markers in treated MCF-7 cells.

## Graphical abstract

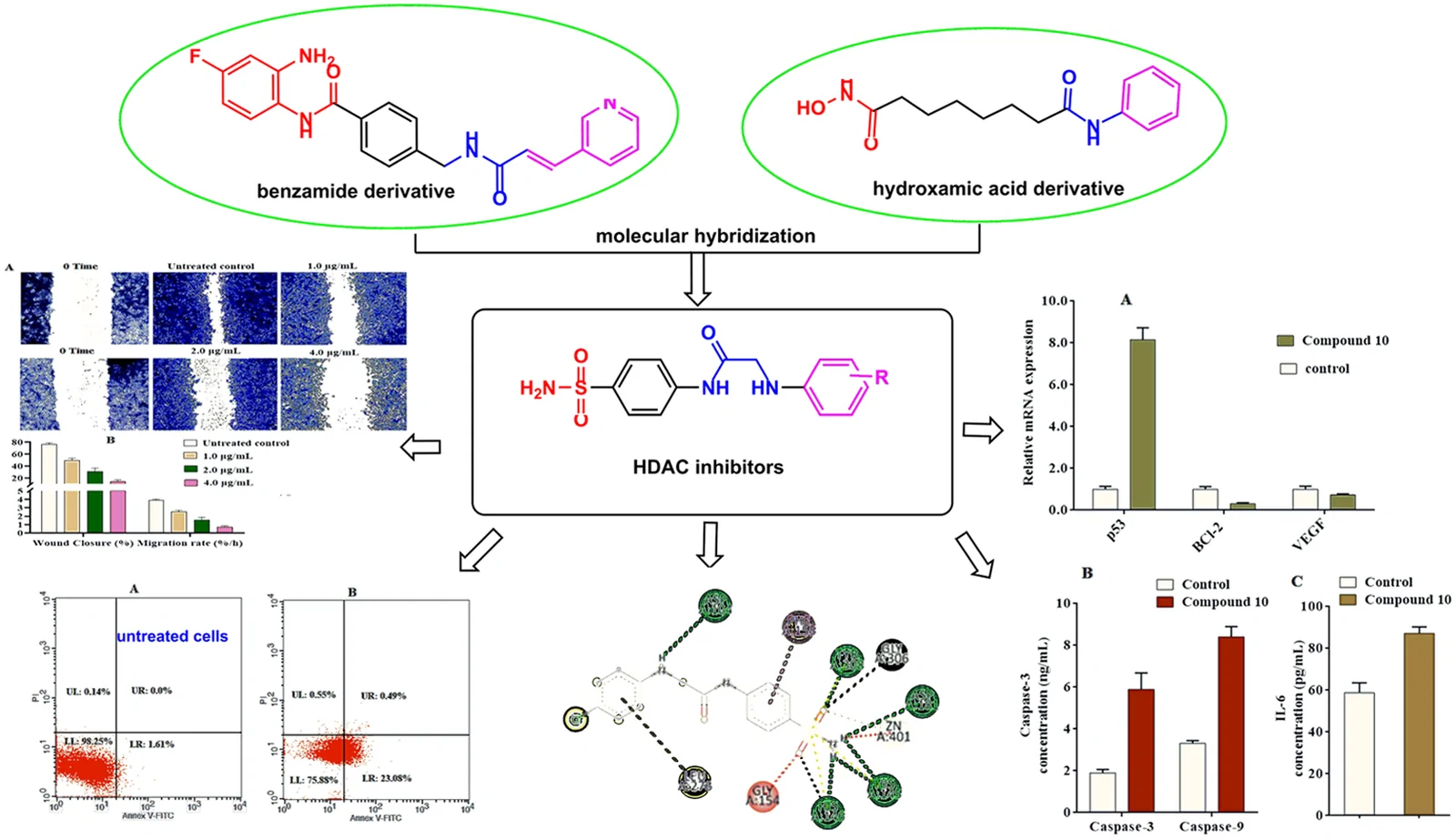

## 1. Introduction

Cancer has been a serious global health threat for decades.^1,2^ However, there are considerable advances in cancer therapy.^3^ Several enzymes emerged as significant targets for cancer treatment.^4^ Such enzymes were proven to be overexpressed in cancer cells rather than normal cells.^5^ So, their inhibition is likely to selectively affect cancer cells, in contrast to traditional chemotherapeutic agents.^6^ One of those effective targeted enzymes is histone deacetylase (HDAC).^7,8^ It was found to be essential for cancer growth and metastasis,^9^ however, the HDAC inhibitors that are currently fully approved by the FDA for cancer treatment are limited to vorinostat and belinostat.^10,11^ They both are hydroxamic acid derivatives.^12^ Meanwhile, no benzamide derivative has been fully approved by the FDA until now.^13^ Those data drew our attention to attempt to develop some new HDAC inhibitors, aiming at discovering a new scaffold and/or effective lead molecules.

HDAC enzymes play crucial roles in cellular growth because they remove acetyl groups from histone lysine residues and thereby regulate chromatin structure.^14,15^ Reversible histone acetylation/deacetylation alters chromatin structure, regulating transcription rate and gene expression.^13^ It was evident that HDAC enzymes stimulate cancer growth and enhance metastasis.^9,16^ The mechanism involved includes promoting angiogenesis and escaping apoptosis.^17,18^ There are approximately eighteen isoforms of HDAC^19^ that are divided into four classes.^20^ Class I comprises the Zn dependent HDACs 1, 2, 3, and 8, and it was found to be highly associated with cancer growth.^21^ Overexpression of HDAC class I in different cancer types such as breast and colon cancer was proven.^22^ Accordingly, they were considered a primary target for cancer treatment as an effective and relatively safe approach.^23,24^ Meanwhile, studies have shown that inhibition of these enzymes showed potent suppression for cancer growth, reducing angiogenesis and inducing cell cycle arrest and cell death.^24–28^

HDAC inhibitors can be categorized into four chemical classes: hydroxamic acid derivatives, benzamides, cyclic peptides, and short chain aliphatic acids.^13,29^ The first two groups (hydroxamic acid derivatives and benzamides) are considered the primary HDAC inhibitors and attracted the attention of researchers.^30^ They both were proven to inhibit HDAC class I.^22,31^ However, the effect of hydroxamic acid derivatives was found to extend to HDAC class II and class IV.^22,31^ Nevertheless, these two classes have common pharmacophores as illustrated in Fig. 1. In the current study we attempted to design new molecules for potential HDAC class I inhibition on the basis of these reported pharmacophores. Additionally, molecular hybridization and properties of functional groups were also involved in the current design. Fig. 1 collectively illustrates the approach to the current design. It can be noticed that four pharmacophores are identified as Zn binding group, hydrophobic linker, polar connecting group, and CAP group. Regarding the Zn binding group, sulfamoyl group as an effective Zn binding group was selected in the current design for such purpose. Sulfonamides are well-known zinc binders in carbonic anhydrase inhibitors. To the best of our knowledge, no previously reported HDAC inhibitor uses a sulfamoyl group as the zinc-binding group. So, evaluation of the impact of the sulfamoyl group in this study is likely to add a new dimension to help discover new potent HDAC inhibitors. The hydrophobic linker was designed as a phenyl ring, while the polar connecting group was constructed to be an aminoacetamide moiety. By looking at the reported HDAC inhibitors, we can notice that the polar connecting group may be amidic group as in vorinostat and tucidinostat or it may be amino group as in panobinostat. We suggest that it may be beneficial to have both groups in the polar connecting group. Finally, the CAP moiety was substituted phenyl ring to examine which of those substituents would be most effective.

**Fig. 1:**
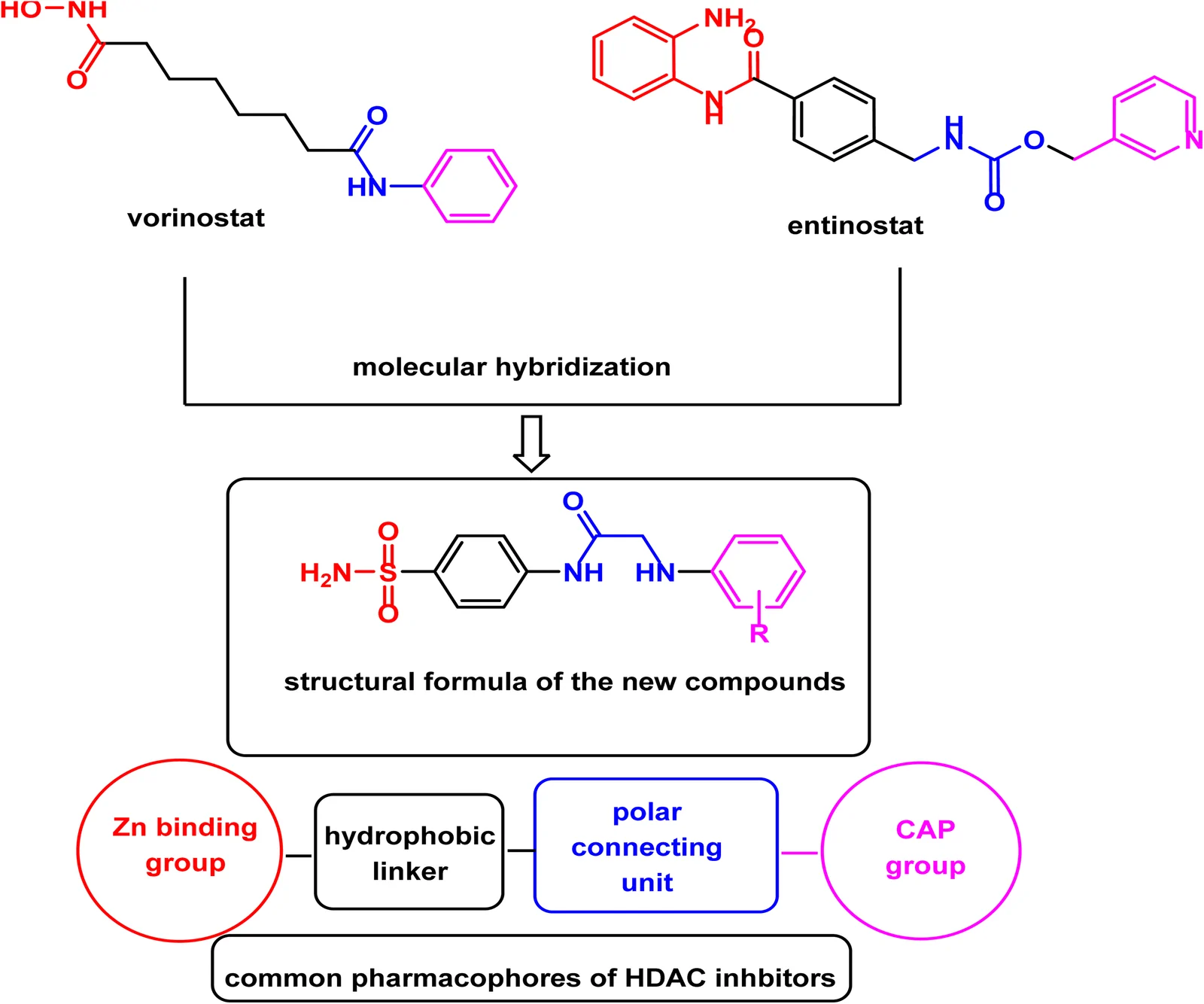
Illustration for the approach to design new HDAC inhibitors on the basis of pharmacophores and molecular hybridization.

## 2. Results and discussion

### 2.1. Chemistry

The designed molecules have been obtained according to the procedure illustrated in Scheme 1. It can be noticed that reaction of sulfanilamide (1) with chloroacetyl chloride (2) afforded the corresponding acetanilide derivative 3, which was then heated with appropriate aromatic amines (4–7), namely 3-aminophenol, 4-aminophenol, 4-chloroaniline, and 4-bromoaniline to give the desired final compounds 8–11.

**Scheme 1:**
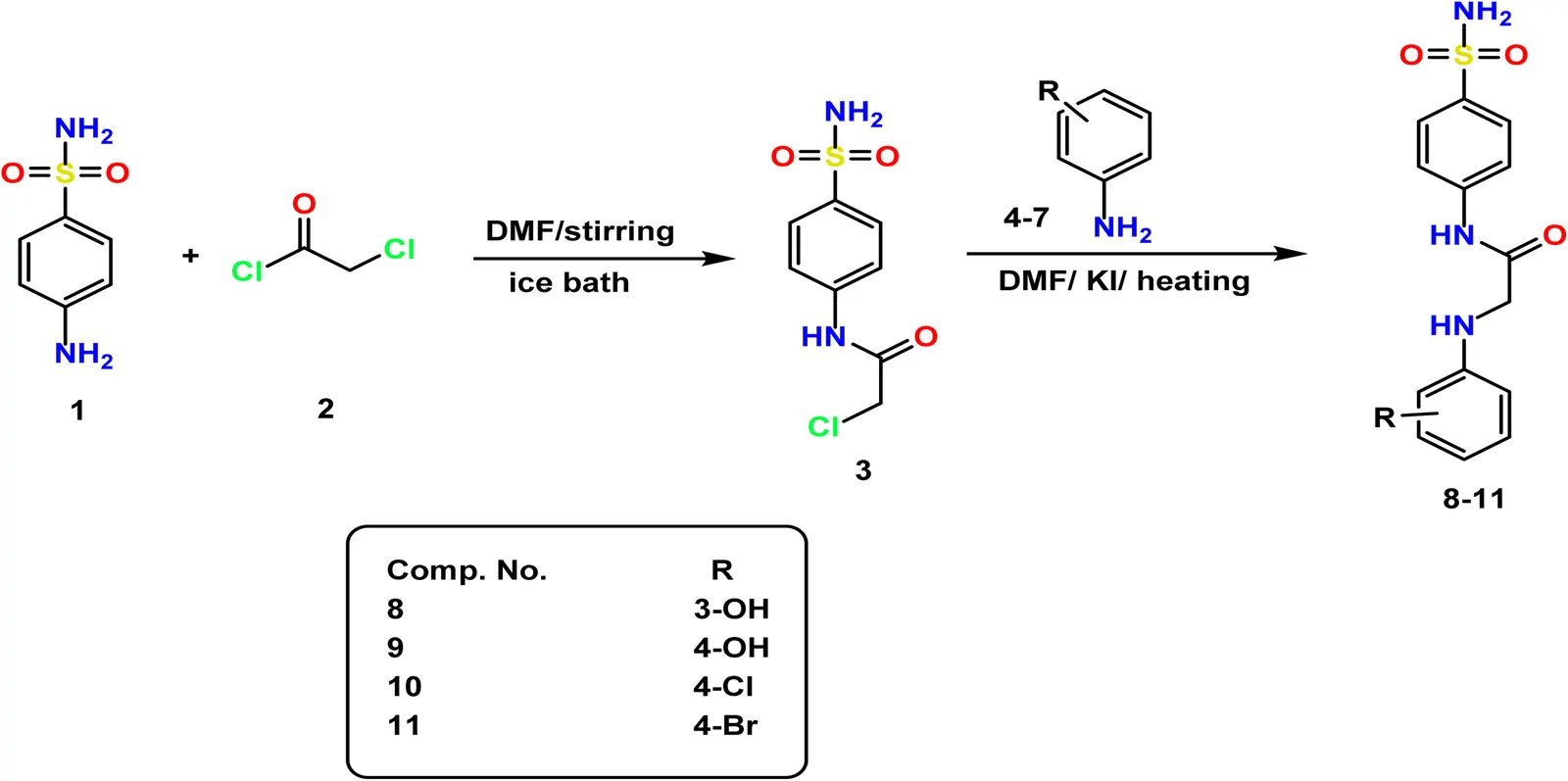
The synthetic method of the final compounds 8–11.

### 2.2. Biological testing

#### 2.2.1. Antiproliferative assay and selectivity profiles

The *in vitro* cytotoxic efficacy of the newly synthesized derivatives (8–11) was evaluated against a panel of human cancer cell lines, including HepG-2 (hepatoma), A549 (lung carcinoma), and MCF-7 (breast carcinoma), alongside normal lung fibroblasts (WI-38). The calculated half-maximal inhibitory/cytotoxic concentrations (IC_50_ and CC_50_) and their corresponding selectivity indices (SI) were compared against the standard chemotherapeutic drug 5-FU (Table 1). All examined compounds demonstrated distinct dose-dependent cytotoxic profiles across the treated concentrations (Fig. 2).

**Table 1:** *In vitro* cytotoxic profiles of CC_50_ values (µM) for normal cell line, IC_50_ (µM) values for cancer cell lines and selectivity indices (SI) of synthesized compounds 8–11 and 5-FU^a^

Comp. no.	WI-38	HepG-2		A549		MCF-7	
	CC_50_	IC_50_	SI	IC_50_	SI	IC_50_	SI
8	76.05 ± 1.52	52.78 ± 2.82	1.44 ± 0.28	47.91 ± 1.33	1.59 ± 0.31	25.46 ± 1.49	2.99 ± 0.50
9	175.6 ± 3.57	37.79 ± 1.72	4.65 ± 0.42	30.07 ± 1.96	5.84 ± 0.54	20.48 ± 1.86	8.57 ± 0.66
10	118.5 ± 2.64	34.71 ± 4.62	3.41 ± 0.39	20.03 ± 2.17	5.92 ± 0.74	13.56 ± 1.36	8.74 ± 0.88
11	133.1 ± 2.54	60.78 ± 2.84	2.19 ± 0.18	67.52 ± 2.82	1.97 ± 0.16	31.39 ± 1.26	4.24 ± 0.30
5-FU	30.99 ± 2.54	18.53 ± 2.23	1.67 ± 0.14	26.61 ± 2.00	1.16 ± 0.09	19.46 ± 1.46	1.59 ± 0.13

a Values are presented as mean ± SD.

**Fig. 2:**
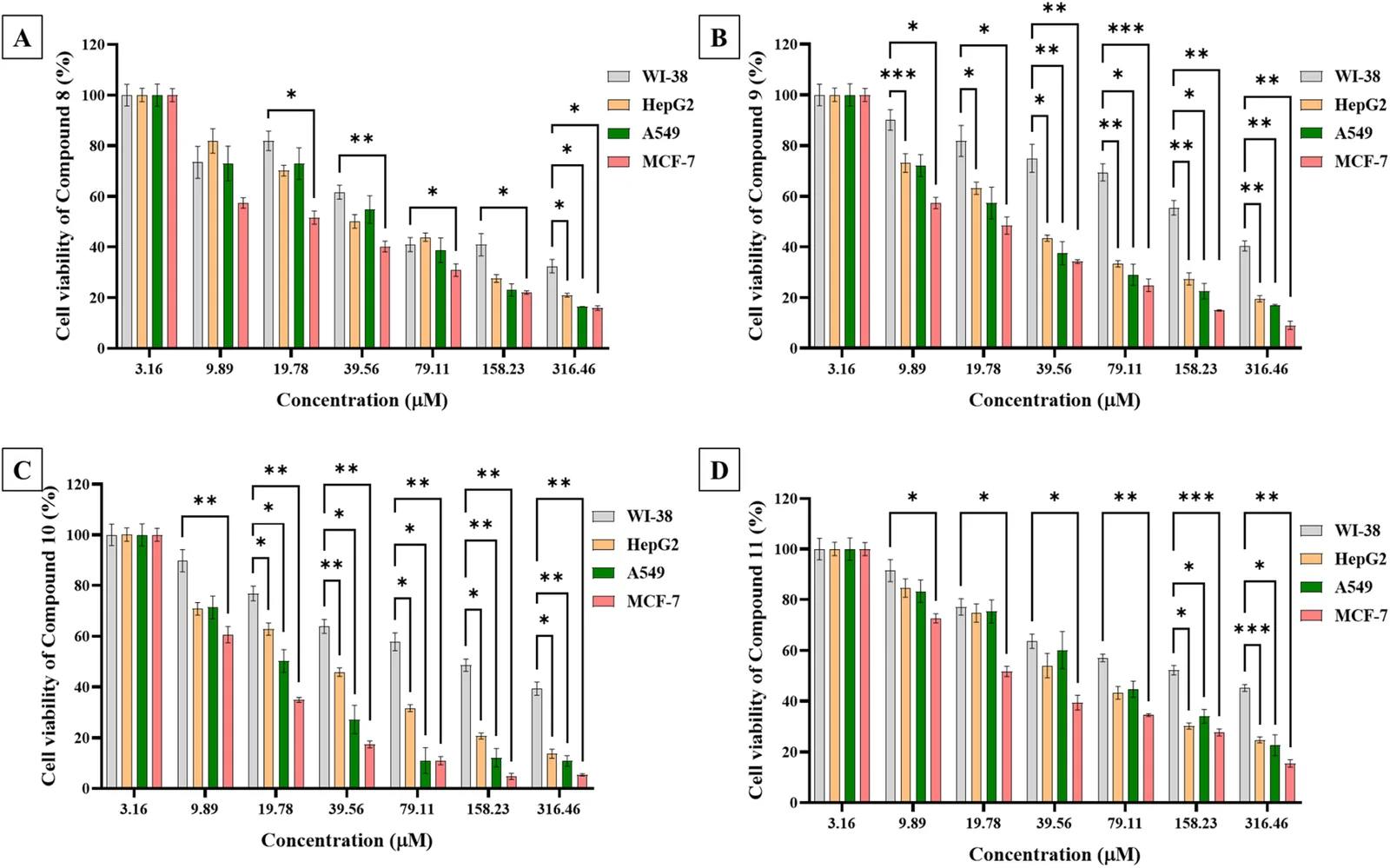
Concentration-dependent effects of the synthesized compounds on the viability of normal human lung fibroblasts (WI-38) and liver (HepG-2), lung (A549), and breast (MCF-7) cancer cell lines after 48 h of treatment. (A) Compound 8, (B) compound 9, (C) compound 10, and (D) compound 11. Values are presented as the mean ± SD of three independent experiments. Statistical analysis was performed using two-way ANOVA. Significance is indicated as: *p < 0.05; **p < 0.01; ***p < 0.001.

Among the synthetic compounds, derivative 10 emerged as the most potent agent across all evaluated cancer cell lines (highest SI in MCF-7 and A549 cells, whereas derivative 9 showed the higher SI in HepG-2 cells). Specifically, MCF-7 cells displayed the highest sensitivity to derivative 10 (IC_50_ = 13.56 ± 1.36 µM; SI = 8.74 ± 0.88). A549 cells were the second most sensitive (IC_50_ = 20.03 ± 2.17 µM; SI = 5.92 ± 0.74), followed by HepG-2 cells (IC_50_ = 34.71 ± 4.62 µM; SI = 3.41 ± 0.39). Crucially, derivative 10 exhibited a markedly greater *in vitro* selectivity toward the tested cancer cells relative to the normal WI-38 cell line than the reference drug 5-FU, which showed comparatively low selectivity indices (SI = 1.16–1.67). Overall, derivative 10 proved to be approximately 2.0- to 5.5-fold more selective toward malignant cells than 5-FU.

Derivative 9 was identified as the second most active derivative against the malignant panel, exhibiting robust cytotoxic effects on MCF-7, A549, and HepG-2 cell lines with IC_50_ values of 20.48 ± 1.86, 30.07 ± 1.96, and 37.79 ± 1.72 µM, respectively. Derivative 8 ranked third in terms of overall anti-proliferative potency, followed by derivative 11, which exhibited the lowest activity among the tested derivatives. Across almost all tested modifications, the MCF-7 cell line consistently demonstrated the greatest vulnerability to treatment compared to A549 and HepG-2 cells. Taken together, the markedly elevated selectivity profiles of these derivatives over 5-FU highlight their promising potential as scaffolds for targeted anticancer drug development.

#### 2.2.2. Structure–activity relationships (SAR)

The data revealed that substitution at the *para* position of the terminal phenyl of the current scaffold is more effective than *meta* position. The most potent substituent at the *para* position is the electron withdrawing chloride in terms of lung and breast carcinoma. This effect may be related to the hydrophobicity of chloride compared to that of hydroxyl group. Whereas the electron donating hydroxyl group (compound 9) showed the highest selectivity index toward hepatoma cells, although compound 10 had the lower IC_50_ value in HepG-2 cells (Table 1). The phenolic hydroxyl represented an acidic group with lower hydrophobicity than chloride and bromide. The bulkiest bromide group at *para* position caused considerable reduction in the activity against all examined cancer cell lines. This may be related to steric effect.

#### 2.2.3. Cell death analysis

Microscopic evaluation revealed pronounced morphological alterations in the treated A549, HepG-2, and MCF-7 cell lines compared to their respective untreated controls (Fig. 3A–C). Upon exposure to the IC_50_ doses of the tested compounds (8–11) and 5-FU as chemotherapy drug, all three cancer lines exhibited classic cytological hallmarks of apoptosis. These changes were characterized primarily by substantial cellular shrinkage, rounding, loss of characteristic monolayer density, and noticeable nuclear condensation. Taken together, these observable phenotypic deviations are consistent with loss of cell viability and cell death; apoptosis was examined specifically for compound 10 using the assays described below.

**Fig. 3:**
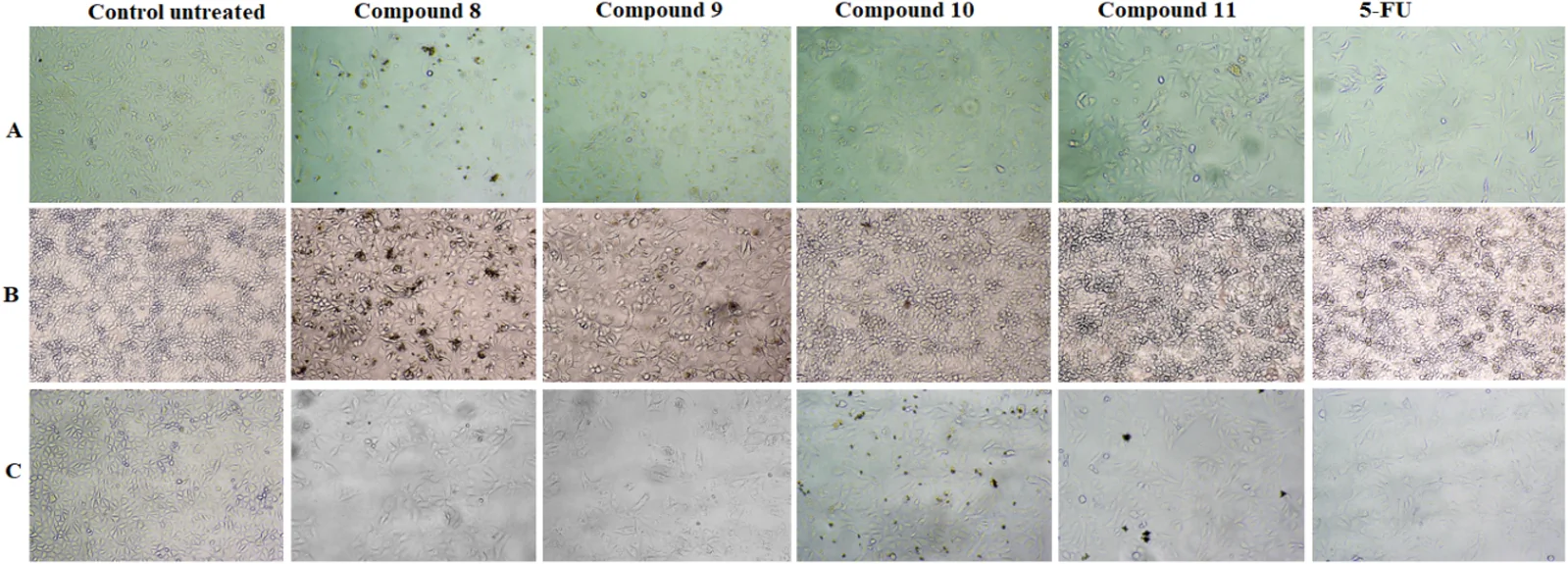
Bright-field micrographs of cells treated with the synthetic compounds (8–11) as compared to untreated cells as control cells and 5-FU treated cells as positive control cells on (A) human lung carcinoma (A549) cell line, and (B) human hepatoma (HepG-2) cell line, and (C) human breast carcinoma (MCF-7) cell line. Scale bar = 100 µm for all panels.

To further elucidate the cellular death mechanisms driven by the chemical modifications, MCF-7 cells were exposed to various concentrations of the compound 10 for 48 h. Single propidium iodide (PI) staining revealed a dose-dependent loss of plasma membrane integrity, while morphological assessment demonstrated characteristic features of apoptosis, most notably chromatin condensation, nuclear shrinkage, and extensive nuclear fragmentation in the treated populations. In contrast, untreated control cells exhibited minimal, uniformly faint background fluorescence, indicating intact nuclear architecture (Fig. 4A). Complementing these findings, dual acridine orange/ethidium bromide (AO/EB) staining provided clear visual confirmation of a concentration-dependent transition through the apoptotic cascade (Fig. 4B). While healthy vehicle control cells emitted a uniform green fluorescence signifying normal membrane integrity and viable cells, treated MCF-7 cells exhibited a distinct shift in color. At lower exposure limits, the appearance of yellow and greenish-orange punctate fluorescence indicated early and late apoptotic populations actively shifting under chemical stress. At the high dose, the cell population progressed entirely to a dense, bright orange-to-reddish fluorescence profile, signaling highly compromised cell membranes characteristic of end-stage apoptotic populations.

**Fig. 4:**
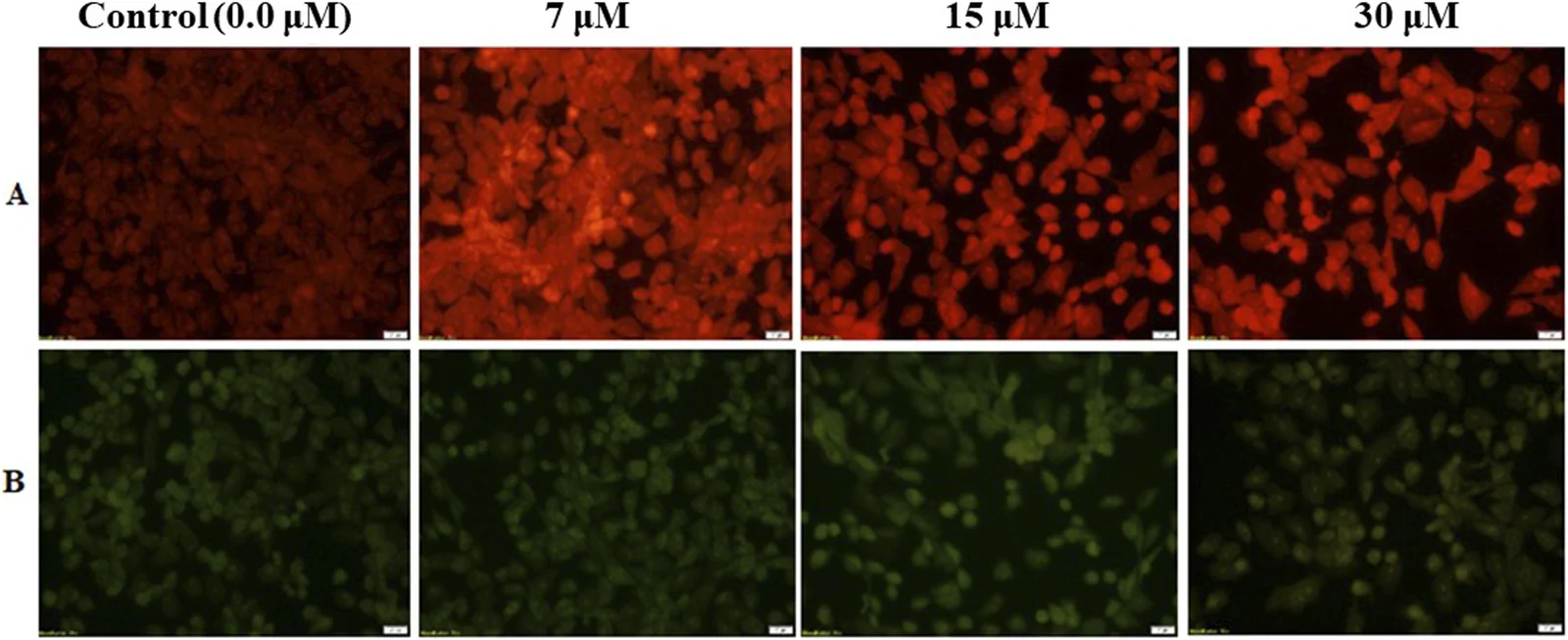
Assessment of the apoptotic effect of the compound 10 on the treated MCF-7 cells at different concentrations (7, 15 and 30 µM) using single PI nuclear staining (A) and double AO/EB nuclear staining (B).

#### 2.2.4. *In vitro* assay of HDAC-1 and HDAC-2 inhibition

To elucidate the potential epigenetic mechanism underlying the observed anticancer profiles, the enzymatic inhibitory activity of the tested compound 10 was evaluated against HDAC-1 and HDAC-2, using the class I-selective HDAC inhibitor entinostat as the positive reference drug (Table 2). Compound 10 exhibited inhibitory efficacy against both target isoforms, yielding an IC_50_ value of 5.66 ± 0.19 µM against HDAC-1 and a lower value of 2.27 ± 0.07 µM against HDAC-2. By comparison, the reference standard entinostat demonstrated superior suppression, with IC_50_ values of 1.04 ± 0.04 µM for HDAC-1 and 1.21 ± 0.04 µM for HDAC-2. These findings clearly indicate that while compound 10 functions with moderate potency relative to the standard control, it retains an affinity for class I HDAC enzymes, particularly the HDAC-2 isoform, which likely drives its downstream apoptotic and anti-proliferative actions in MCF-7 cells.

**Table 2:** *In vitro* inhibitory profiles (IC_50_ values) of compound 10 and entinostat against HDAC-1 and HDAC-2 enzymes^a^

Tested sample	HDAC-1 IC_50_ (µM)	HDAC-2 IC_50_ (µM)
Compound 10	5.66 ± 0.19	2.27 ± 0.07
Entinostat	1.04 ± 0.04	1.21 ± 0.04

a Values are presented as mean ± SD.

#### 2.2.5. Anti-migration activity

The anti-migratory activity of compound 10 was assessed in MCF-7 cells using a scratch wound-healing assay. As shown in Fig. 5A, untreated cells exhibited rapid wound closure within 48 h, reflecting their high migratory capacity. In contrast, treatment with compound 10 inhibited wound closure in a concentration-dependent manner, indicating reduced cell motility. Quantitative analysis (Fig. 5B) demonstrated that wound closure was reduced from 76.59% in control cells to 49.52%, 31.16%, and 14.15% following treatment with 3, 7, 15 µM, respectively. Correspondingly, migration rates (Fig. 5C) declined from 3.91%/h in untreated cells to 2.53%/h, 1.59%/h, and 0.72%/h. These findings highlight the potent migration-suppressive effect of compound 10, potentially mediated through disruption of cytoskeletal organization and inhibition of signaling pathways involved in epithelial–mesenchymal transition and metastatic progression.

**Fig. 5:**
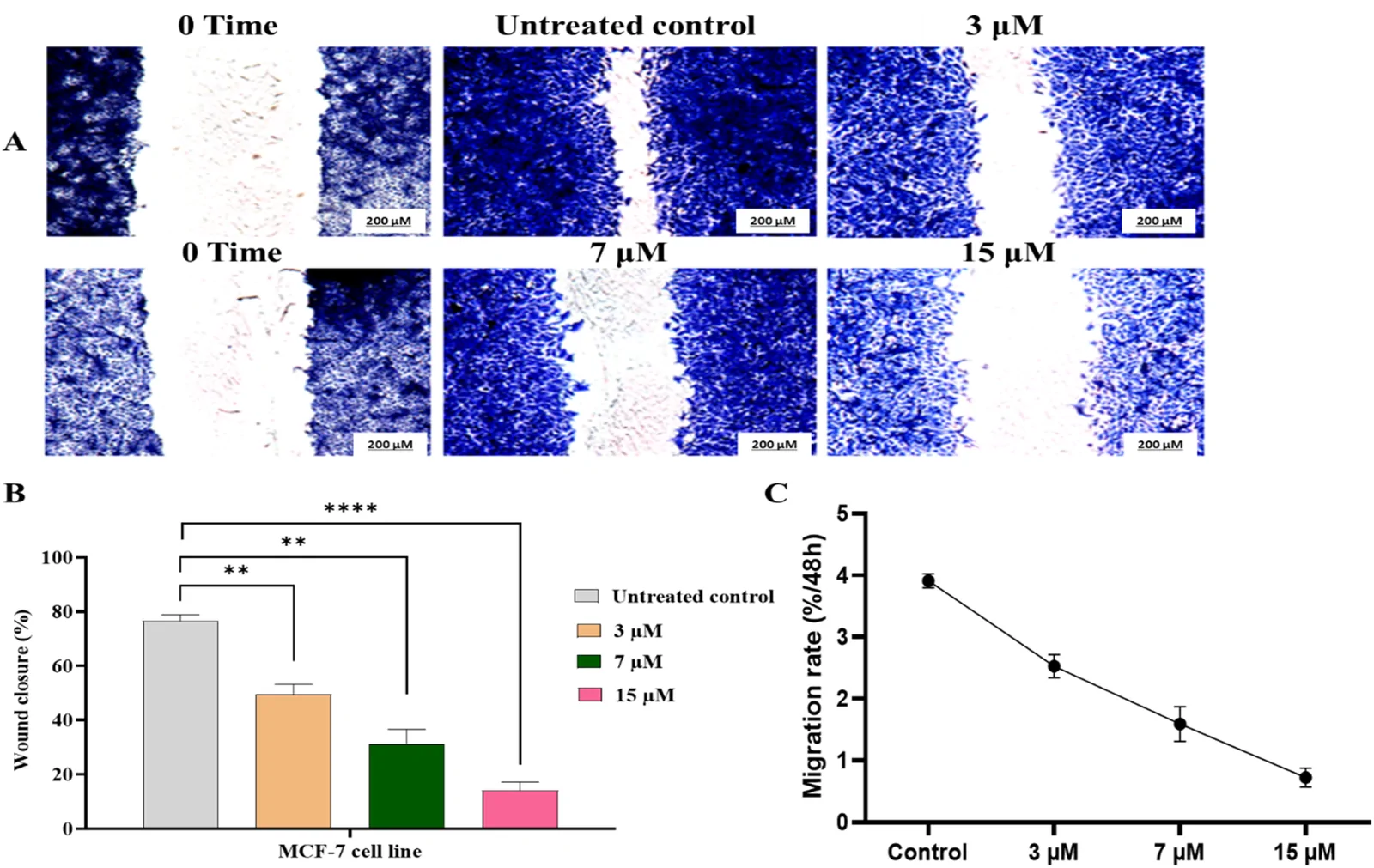
Inhibition of MCF-7 cell migration following treatment with compound 10. (A) Representative images from the scratch assay showing suppression of MCF-7 cell migration after 48 h exposure to compound 10 at concentrations of 3, 7, 15 µM compared to untreated control cells and captured cells at 0 time. (B) Quantification of wound closure percentages and (C) migration rates relative to untreated control cells. Data are presented as mean ± SD (*n* = 3 independent experiments). Statistical significance was assessed using Brown–Forsythe and Welch ANOVA, followed by Dunnett's T3 multiple comparisons test. A *p*-value < 0.05 was considered statistically significant.

#### 2.2.6. Annexin V-FITC apoptosis assay

To precisely quantify the proportions of apoptotic and necrotic cells induced by treatment, an Annexin V-FITC/PI dual-staining assay was performed on MCF-7 cells following exposure to the IC_50_ concentration of compound 10 for 48 h. As detailed in Table 3 and Fig. 6, the experimental results demonstrate that compound 10 shifts the cellular profile heavily toward programmed cell death pathways rather than non-specific necrosis. Compared to the untreated control cells, which exhibited a baseline total apoptotic population of 1.61%, treatment with compound 10 markedly elevated total apoptosis to 23.57%. Specifically, the early apoptotic cell population increased from 1.61% in the control group to 23.08% in the treated group. Meanwhile, late apoptotic cells underwent a minor increase from 0.0% to 0.49%. Taken together, compound 10 elicited an approximately 14.64-fold increase in the total number of apoptotic cells relative to the untreated control cells. Conversely, the necrotic cell fraction showed only a negligible shift, moving from 0.14% in control cultures to 0.55% at the tested concentration. These results indicate that the anti-proliferative mechanism of compound 10 is predominantly associated with apoptosis rather than necrosis at the tested concentration and time point.

**Table 3:** Apoptosis and necrosis rates (%) of MCF-7 cells treated with compound 10 at IC_50_ concentration compared to control untreated cells

Sample	Apoptosis			Necrosis
	Total	Early	Late	
Untreated cells	1.61	1.61	0.0	0.14
Treated cells	23.57	23.08	0.49	0.55

**Fig. 6:**
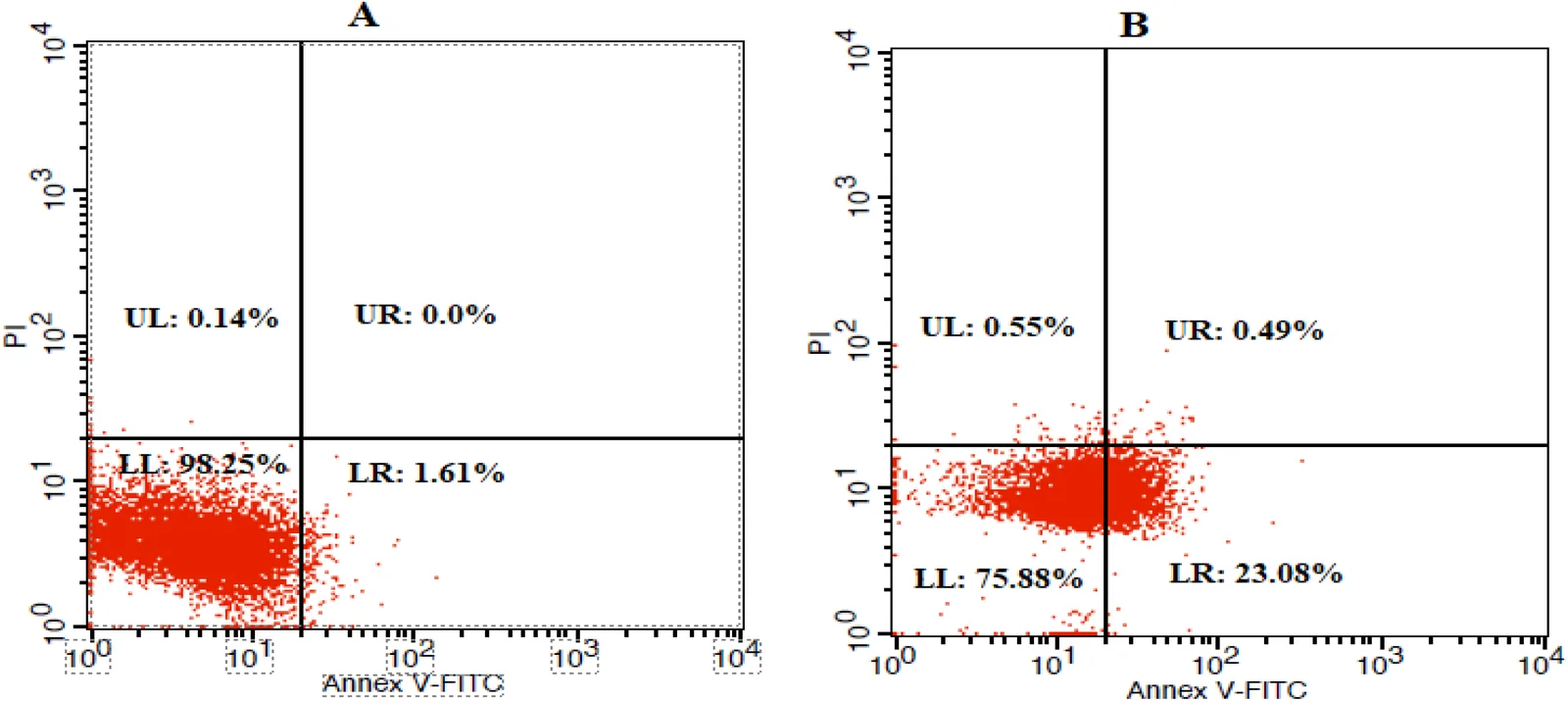
Apoptotic effect of compound 10 on MCF-7 cells. (A) Untreated control cells; (B) apoptosis rates of the treated MCF-7 cells at the IC_50_ dose.

#### 2.2.7. Gene expression and protein-level analyses

To investigate the molecular responses to compound 10, the expression of p53, Bcl-2, and VEGF was assessed by qPCR, while IL-6, and caspase-9 levels were measured by ELISA in MCF-7 breast cancer cells treated at the IC_50_ concentration of compound 10, with 5-FU as a comparator (Fig. 7). Quantitative analysis showed that treatment with compound 10 and 5-FU reduced the expression of the anti-apoptotic gene *Bcl-2* by 3.23-fold and 1.73-fold, respectively, relative to untreated control cells. In contrast, compound 10 increased the expression of the pro-apoptotic tumor suppressor gene *p53* 8.14-fold relative to untreated control cells and 2.51-fold relative to the 5-FU-treated group (Fig. 7A). These changes are consistent with a shift toward a pro-apoptotic gene-expression profile following treatment with compound 10. Furthermore, expression of the angiogenic factor *VEGF* decreased 1.37-fold and 2.27-fold following treatment with compound 10 and 5-FU, respectively, relative to untreated control cells. These findings suggest that compound 10 may influence both apoptotic and angiogenesis-related pathways. To further investigate the apoptotic response, and caspase-9 levels were measured by ELISA in MCF-7 cells following 48 hour exposure to compound 10 (Fig. 7B). Similarly, caspase-9 levels increased 2.54-fold, from 3.31 ng mL^−1^ to 8.40 ng mL^−1^. The increase in caspase-9 concentration supports the involvement of apoptosis in the cellular response to compound 10. IL-6 concentrations also increased 1.48-fold, from 58.69 pg mL^−1^ in untreated control cells to 87.05 pg mL^−1^ following treatment with compound 10 (Fig. 7C). However, IL-6 levels decreased following 5-FU treatment. Although IL-6 levels increased following treatment with compound 10, this response may reflect treatment-induced cellular stress or an inflammatory response. Given the concurrent upregulation of *p53* and the increased caspase-9 level, together with the downregulation of *Bcl-2*, the overall molecular profile remains consistent with apoptosis induction.^32^

**Fig. 7:**
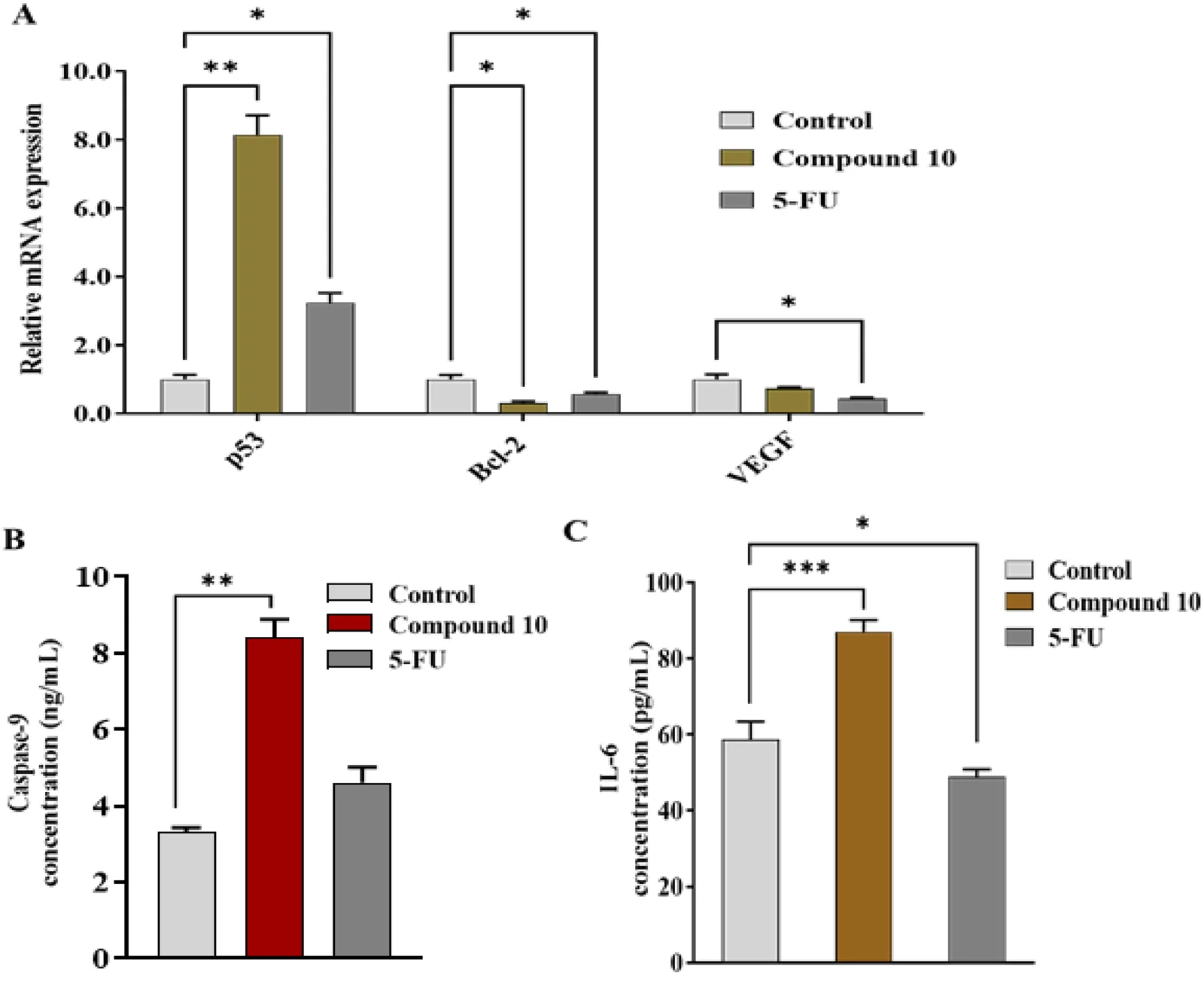
Effect of compound 10 on apoptosis-related markers in MCF-7 cells after 48 h of treatment with the IC_50_ concentration of compound 10 or 5-FU. (A) Relative mRNA expression of p53, Bcl-2 and VEGF determined by qPCR. (B) Caspase-9 level and (C) IL-6 level measured by ELISA. Data are mean ± SD of three independent experiments. Statistical analysis: two-way ANOVA followed by Dunnett test for (A); one-way ANOVA for (B and C). *p < 0.05, **p < 0.01, ***p < 0.001 *vs.* untreated control. Comparisons without an asterisk were not significant (ns).

### 2.3. Docking study

It was interesting to get insights into the suggested binding pattern of the current new scaffold with HDAC. So, the HDAC-2 structure (ID: 4LXZ) was downloaded from Protein Data Bank with the co-crystallized ligand vorinostat. Redocking of vorinostat showed RMSD of 1.3 Å, indicating the validation of docking protocol. The new compounds were prepared and docked into the isolated pocket. The docking results showed the ability of the new derivatives to bind firmly with the receptor in a manner similar to that of vorinostat as discussed below. This finding revealed the significance of the designed scaffold as a lead for discovery of new HDAC inhibitors.

The binding mode of vorinostat showed different types of bonds as shown in Fig. 8. We can notice an essential bond with Zn ion *via* the hydroxamate group. Meanwhile, there are hydrogen bonds with the essential residues His145, His146, Tyr308 (*via* the hydroxamate group) and Asp104 (*via* the polar connecting amide group). Furthermore, hydrophobic interactions with the amino acids Phe155, Phe210 and Leu276 can also be noticed.

**Fig. 8:**
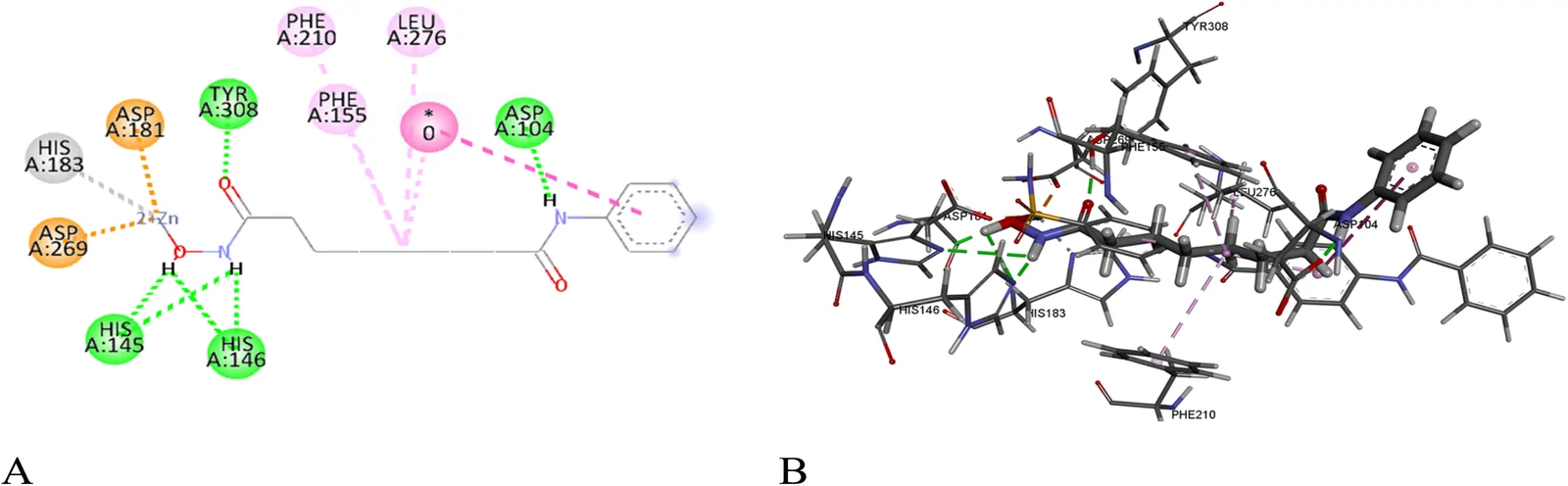
Binding pattern of vorinostat with HDAC-2 receptor; (A) 2D image, (B) 3D image.

Compound 8 revealed a binding mode very similar to that of vorinostat, where the sulfonamide group bonded to Zn ion *via* sulfonamide group as illustrated in Fig. 9. Similarly, there are hydrogen bonds with the essential amino acids His145, His146, Tyr308 (*via* the sulfonamide group), and Asp104 (*via* the polar connecting amino group). An additional hydrogen bond with Arg275 can also be observed. There is also pi–pi interaction with Phe155, pi–alkyl interaction with Leu276, and pi–sulfur interactions with His145, His146, Tyr308.

**Fig. 9:**
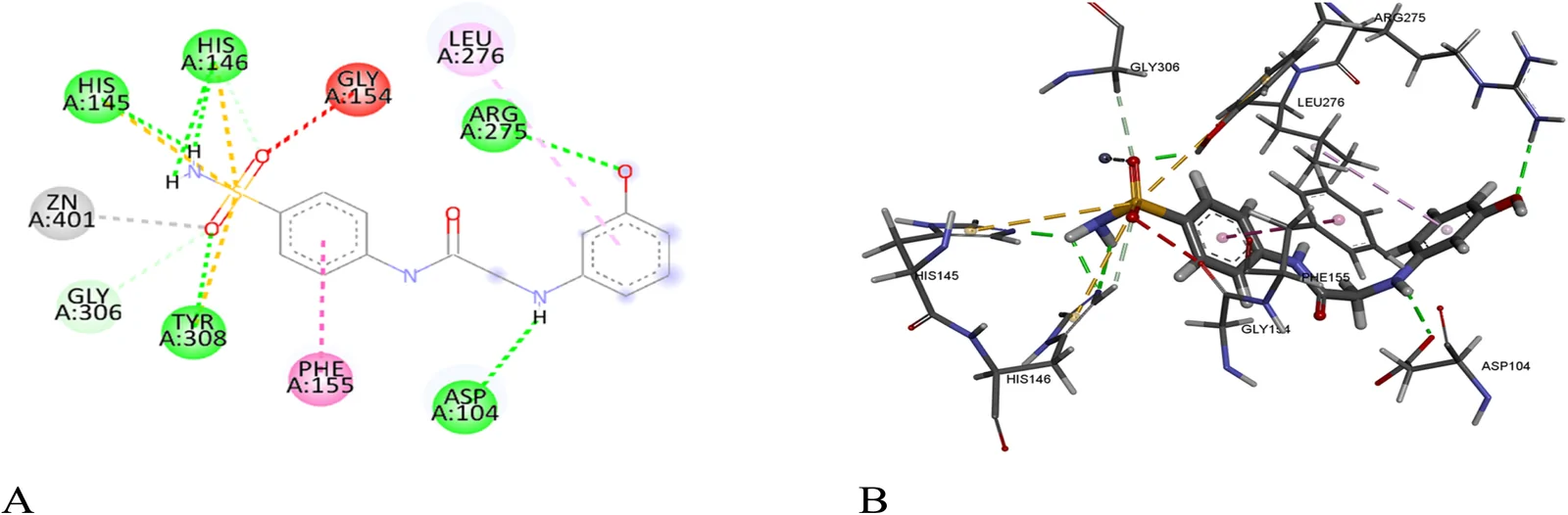
(A) 2D illustration of binding mode of compound 8; (B) 3D illustration of binding mode of compound 8.

A similar binding pattern for compound 10 was illustrated in Fig. 10. We can notice that the sulfonamide group showed binding with Zn ion and hydrogen bonds with the essential residues His145, His146, and Tyr308. A further hydrogen bond with Asp181 was also obtained. The essential hydrogen bond between Asp104 and the connecting polar group occurred efficiently. There are also pi–pi interaction with Phe155, pi–alkyl interaction with Leu276, and pi–sulfur interactions with His145, His146, Tyr308.

**Fig. 10:**
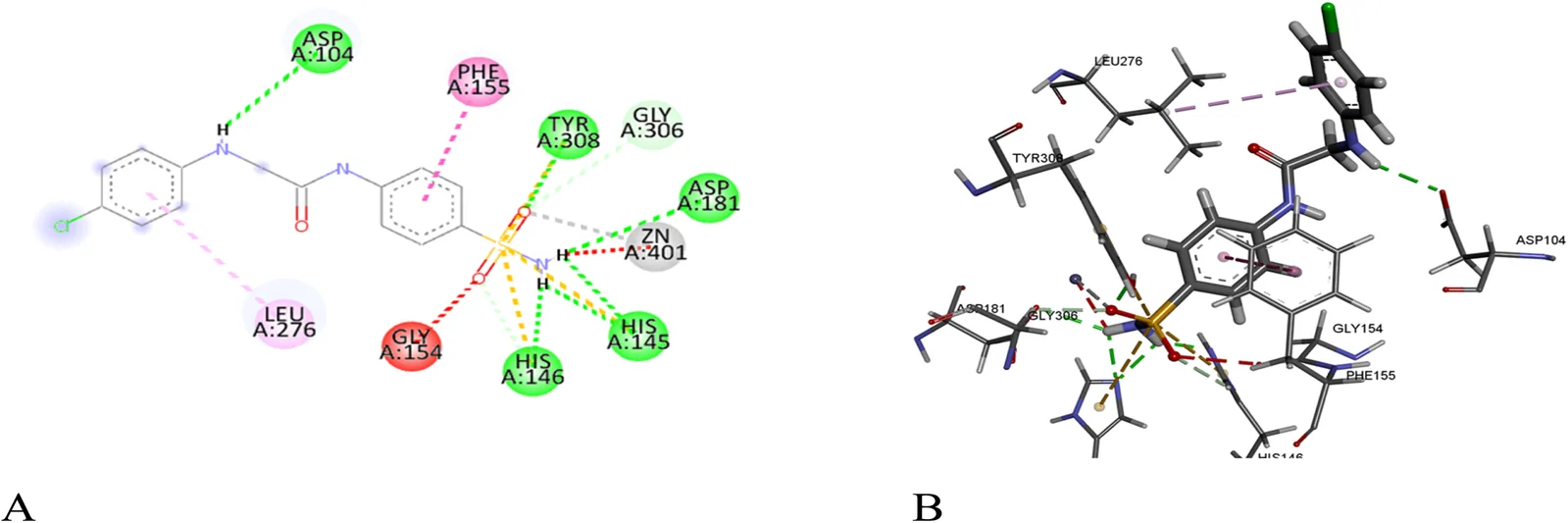
Binding mode of compound 10 to HDAC-2; (A) 2D illustration, (B) 3D illustration.

Regarding the binding patterns of compound 9 and 11, they were found to be very close to those of vorinostat, compound 8 and compound 10 as illustrated in the SI.

The binding affinities of the new derivatives and vorinostat to HDAC-2 are outlined in Table 4. As can be noticed the descending order of the new molecules was found to be compound 10, 8, 11, and 9. The score of compound 10 was the most effective among the new candidates. However, vorinostat score was better than those of the new derivatives according to obtained results.

**Table 4:** Binding affinities (kcal mol^−1^) of the new derivatives and vorinostat to HDAC-2

Compound no.	Vorinostat	8	9	10	11
Binding energy	−6.68	−4.33	−4.02	−4.80	−4.17

## 3. Conclusion

On the basis of the *in vitro* biological results, the current study suggests that compound 10, as an HDAC inhibitor, is a lead scaffold worthy of further optimization toward anticancer agents. It is recommended to utilize the current sulfonamide scaffold for the future discovery of new HDAC inhibitors. The docking data revealed the ability of sulfonamide not only to bind to Zn ion, but also to show different types of effective interactions with essential amino acids in the HDAC-2 pocket. Biologically, the molecules of this scaffold showed promising antiproliferative activity, particularly compound 10. Interestingly, compound 10 exhibited greater *in vitro* selectivity toward the tested cancer cells relative to the normal WI-38 cell line compared with 5-FU. Additionally, it was associated with apoptosis (Annexin V-FITC/PI) and with altered levels of apoptosis-related markers. Furthermore, it inhibited HDAC-2 in a cell-free assay and showed a plausible binding mode in the docking model. However, a limitation of this study is that complementary mechanistic investigations, including histone acetylation and cell-cycle analyses, were not performed; cellular target engagement, isoform and carbonic anhydrase selectivity, and *in vivo* efficacy also remain to be evaluated.

## 4. Materials and methods

### 4.1. Chemistry

The data of solvents, chemical reagents and instruments used in the current work are described in the SI.

#### 4.1.1. General synthetic method of final compounds (8–11)

A mixture of equimolar amounts of 2-chloro-*N*-(4-sulfamoylphenyl)acetamide 3 and an appropriate aromatic amine (4–7), namely 3-aminophenol, 4-aminophenol, 4-chloroaniline, and 4-bromoaniline in DMF was heated for 6 hours in a water bath in the presence a catalytic amount of KI. The reaction mixture was allowed to cool then poured dropwise into ice water with stirring. The obtained precipitate was filtered off, dried and crystallized from ethanol to afford the corresponding final compounds 8–11.

##### 4.1.1.1. 2-((3-Hydroxyphenyl)amino)-*N*-(4-sulfamoylphenyl)acetamide (8)

Color: brown solid; yield: 79.50%; mp: 210–212 °C; ^1^H NMR (DMSO-d_6_): *δ* 10.97 (s, 1H, CONH), 9.01 (s, 1H, OH), 8.41 (s, 1H, NH), 7.85–7.74 (m, 4H, Ar-H), 7.66–7.54 (m, 3H, Ar-H), 7.36–7.27 (m, 3H, Ar-H & NH_2_), 5.01 (s, 2H, CH_2_); ^13^C NMR (DMSO-d_6_): *δ* 167.99, 154.45, 150.01, 139.32, 136.80, 129.67, 127.32, 122.61, 121.97, 119.51, 113.47, 70.35; anal. calcd for C_14_H_15_N_3_O_4_S (321.35): C, 52.33; H, 4.71; N, 13.08. Found: C, 52.51; H, 4.86; N, 13.29.

##### 4.1.1.2. 2-((4-Hydroxyphenyl)amino)-*N*-(4-sulfamoylphenyl)acetamide (9)

Color: brown solid; yield: 82.60%; mp: 228–230 °C; ^1^H NMR (DMSOd_6_): *δ* 11.14 (s, 1H, CONH), 10.60 (s, 1H, OH), 8.06 (s, 1H, NH), 8.02 (d, *J* = 8.32 Hz, 1H, Ar-H), 7.86–7.80 (m, 3H, Ar-H), 7.42 (d, *J* = 8.24 Hz, 1H, Ar-H), 7.28 (s, 2H, NH_2_), 6.87 (d, *J* = 8.24 Hz, 1H, Ar-H), 6.67 (d, *J* = 8.56 Hz, 1H, Ar-H), 6.42 (d, *J* = 8.44 Hz, 1H, Ar-H), 4.31 (s, 2H, CH_2_); ^13^C NMR (DMSO-d6): *δ* 171.88, 158.95, 150.78, 127.38, 127.09, 124.52, 120.13, 119.35, 116.46, 57.72; anal. calcd for C_14_H_15_N_3_O_4_S (321.35): C, 52.33; H, 4.71; N, 13.08. Found: C, 52.49; H, 4.85; N, 13.26.

##### 4.1.1.3. 2-((4-Chlorophenyl)amino)-*N*-(4-sulfamoylphenyl)acetamide (10)

Color: gray solid; yield: 83.60%; mp: 256–258 °C; ^1^H NMR (DMSO-d_6_): *δ* 10.35 (s, 1H, CONH), 7.82–7.74 (m, 4H, Ar-H), 7.25 (s, 2H, NH_2_) 7.14–7.10 (d, *J* = 8.72 Hz, 2H, Ar-H), 6.63–6.57 (d, *J* = 8.72 Hz, 2H, Ar-H), 6.23 (t, *J* = 5.96 Hz, IH, NH), 3.92 (s, 2H, CH_2_); ^13^C NMR (DMSO-d_6_): *δ* 170.11, 147.74, 142.17, 138.90, 129.05, 127.16, 120.18, 119.27, 114.16, 47.61; anal. calcd for C_14_H_14_ClN_3_O_3_S (339.79): C, 49.49; H, 4.15; N, 12.37. Found: C, 49.62; H, 4.31; N, 12.49.

##### 4.1.1.4. 2-((4-Bromophenyl)amino)-*N*-(4-sulfamoylphenyl)acetamide (11)

Color: yellow solid; yield: 84.00%; mp: 280–282 °C; ^1^H NMR (DMSO-d_6_): *δ* 10.81 (s, 1H, CONH), 8.04–7.74 (m, 8H, Ar-H), 7.29 (s, 2H, NH_2_), 6.70 (s, 1H, NH), 4.20 (s, 2H, CH_2_); ^13^C NMR (DMSO-d_6_): *δ* 168.79, 154.88, 142.09, 139.00, 136.87, 127.23, 126.54, 119.29, 112.86, 46.60; anal. calcd for C_14_H_14_BrN_3_O_3_S (384.25): C, 43.76; H, 3.67; N, 10.94. Found: C, 43.94; H, 3.75; N, 11.22.

### 4.2. Biological testing

#### 4.2.1. *In vitro* anti-proliferative assay

MTT assay, according to the reported method, has been applied to examine the new derivatives against three cancer cell lines namely: HepG-2 (hepatoma), A549 (lung carcinoma), and MCF-7 (breast carcinoma) (see the SI for details).^33–35^

#### 4.2.2. Cell death assessment

Fluorescence microscopy was applied to investigate early and late apoptosis according to the methods^36,37^ supplied in the SI.

#### 4.2.3. *In vitro* HDAC-1 and HDAC-2 inhibition assay

HDAC-1 and HDAC-2 inhibitory potential of the synthesized compound 10 was evaluated using a commercial fluorometric HDAC inhibitor screening kit (BPS Bioscience, USA) in accordance with the manufacturer's instructions^22^ (see SI).

#### 4.2.4. Annexin V-FITC apoptosis assay

To quantify the percentages of apoptotic and necrotic cell populations, an Annexin V-fluorescein isothiocyanate (FITC)/propidium iodide (PI) dual-staining assay was performed (see SI).^38^

#### 4.2.5. Gene expression testing and effect on caspase-9 and secreted IL-6 levels

According to the method described in the SI, p53, Bcl-2, VEGF, caspase-9, and IL-6 expression levels have been determined.^39^

### 4.3. Molecular docking

Docking protocol^40–47^ of the new derivatives into the X-ray crystal structure of human HDAC-2 (PDB ID: 4LXZ, downloaded from the RCSB Protein Data Bank), is described in the SI.

### 4.4. Statistical analysis

All experiments were performed independently in triplicate, and results are expressed as the mean ± standard deviation (SD). Statistical analyses were performed using GraphPad Prism version 9.0. One-way or two-way analysis of variance (ANOVA) was used, as appropriate to the experimental design and data structure, followed by the corresponding *post hoc* multiple-comparison test. The statistical test and *post hoc* procedure used for each experiment are specified in the relevant figure legends. A *P* value < 0.05 was considered statistically significant. The selectivity index (SI) was calculated as CC_50_ (WI-38)/IC_50_ (cancer cell line).

## Conflicts of interest

The authors have no competing interests relevant to this work to declare.

## Supplementary Material

RA-OLF-D6RA05385H-s001

RA-OLF-D6RA05385H-s002

## Data Availability

The data supporting this article have been included as part of the supplementary information (SI). Supplementary information: a chemistry part, biological data, and molecular docking supporting materials. See DOI: https://doi.org/10.1039/d6ra05385h.
