# Supplementary material for: New sulfonamide derivatives as HDAC inhibitors and apoptosis inducers: design, synthesis, in silico study and anticancer evaluation
Source: RSC Adv. 2026 Oct 27. Online ahead of print. doi: 10.1039/d6ra05385h (PMC13711385; doi:10.1039/d6ra05385h)
Supplement: Supplementary file 1 [file RA-OLF-D6RA05385H-s002.pdf]

This journal is © The Royal Society of Chemistry

Chemistry part

All solvents and chemicals were obtained from commercial suppliers and used without further purification. Reactions were monitored by TLC (Kieselgel 60 F254 precoated plates, E. Merck, Germany), and the spots were detected by exposure to UV lamp at λ 254 nm. All melting points were determined on a Gallen lamp melting point apparatus and were uncorrected. The ^{1}H NMR and ^{13}C NMR spectra were determined in deuterated dimethyl sulfoxide (DMSO-*d*6) depending on the compound's solubility. The NMR instrument is BRUKER 400 MHZ-NMR spectro-photometer at Faculty of Pharmacy, Cairo University, Egypt. Chemical shifts δ are reported in parts per million (ppm) downfield from tetramethylsilane (TMS) internal standard. Elemental microanalyses were performed on a CHN analyzer at the Regional Center for Mycology and Biotechnology, Faculty of Science, Al-Azhar University, Egypt.

^{1}HNMR and ^{13}CNMR charts of the new derivatives

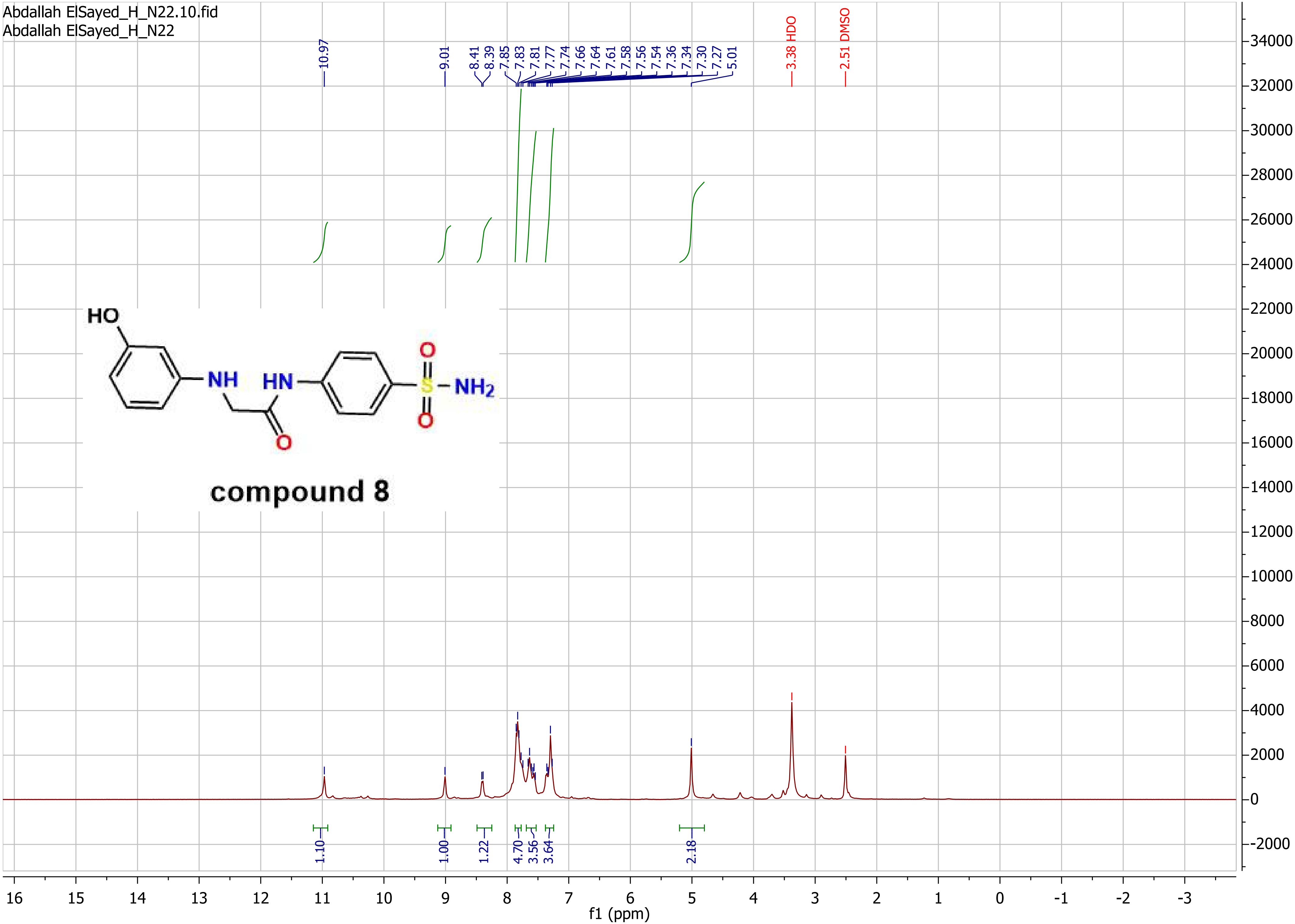

Abdallah ElSayed_H_N22.10.fid
Abdallah ElSayed_H_N22
10.97
9.01
8.41
8.39
7.85
7.83
7.81
7.77
7.74
7.66
7.64
7.61
7.58
7.56
7.54
7.36
7.34
7.30
7.27
5.01
3.38 HDO
2.51 DMSO
HO
NH
HN
S
O
NH2
compound 8
1.10
1.00
1.22
4.70
3.56
3.64
2.18
f1 (ppm)

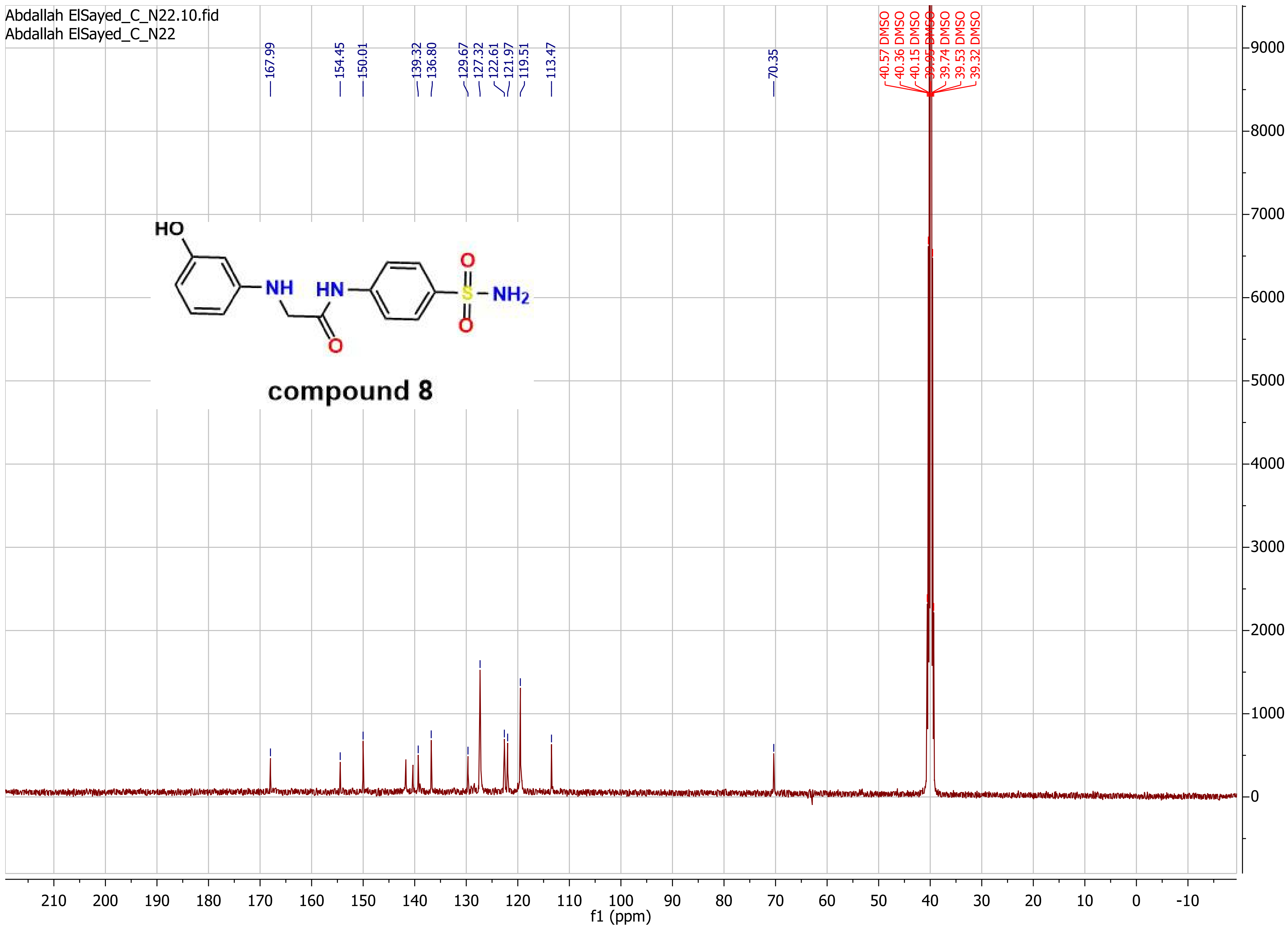
Abdallah ElSayed_C_N22.10.fid
Abdallah ElSayed_C_N22
167.99
154.45
150.01
139.32
136.80
129.67
127.32
122.61
121.97
119.51
113.47
70.35
40.57 DMSO
40.36 DMSO
40.15 DMSO
39.95 DMSO
39.74 DMSO
39.53 DMSO
39.32 DMSO
HO
NH
HN
O
S
NH2
O
O
compound 8
f1 (ppm)

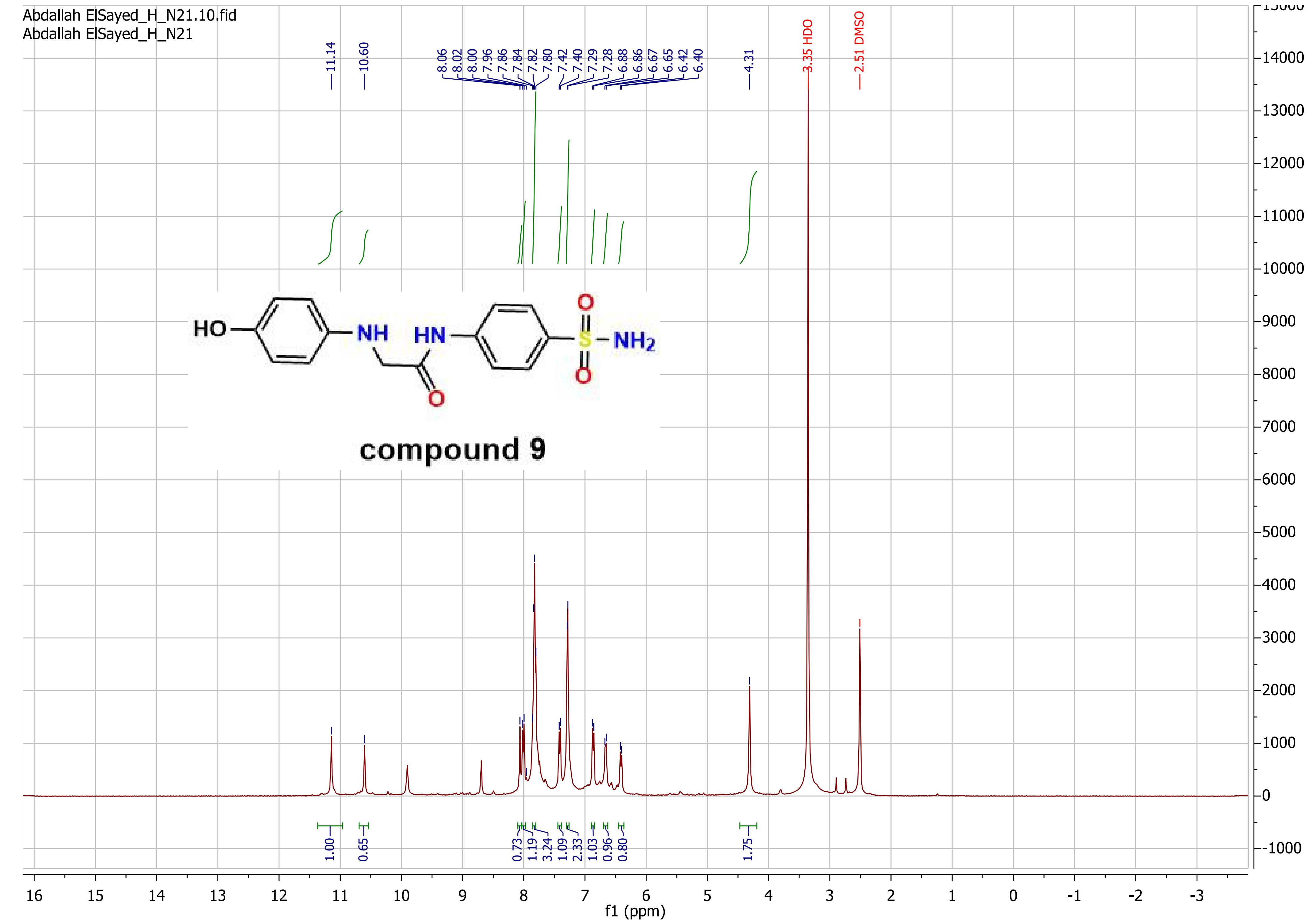
Abdallah ElSayed_H_N21.10.fid
Abdallah ElSayed_H_N21
11.14
10.60
8.06
8.02
8.00
7.96
7.86
7.84
7.82
7.80
7.42
7.40
7.29
7.28
6.88
6.86
6.67
6.65
6.42
6.40
4.31
3.35 HDO
2.51 DMSO
HO
NH
HN
S
O
NH2
compound 9
1.00
0.65
0.73
1.19
3.24
1.09
2.33
1.03
0.96
0.80
1.75
f1 (ppm)

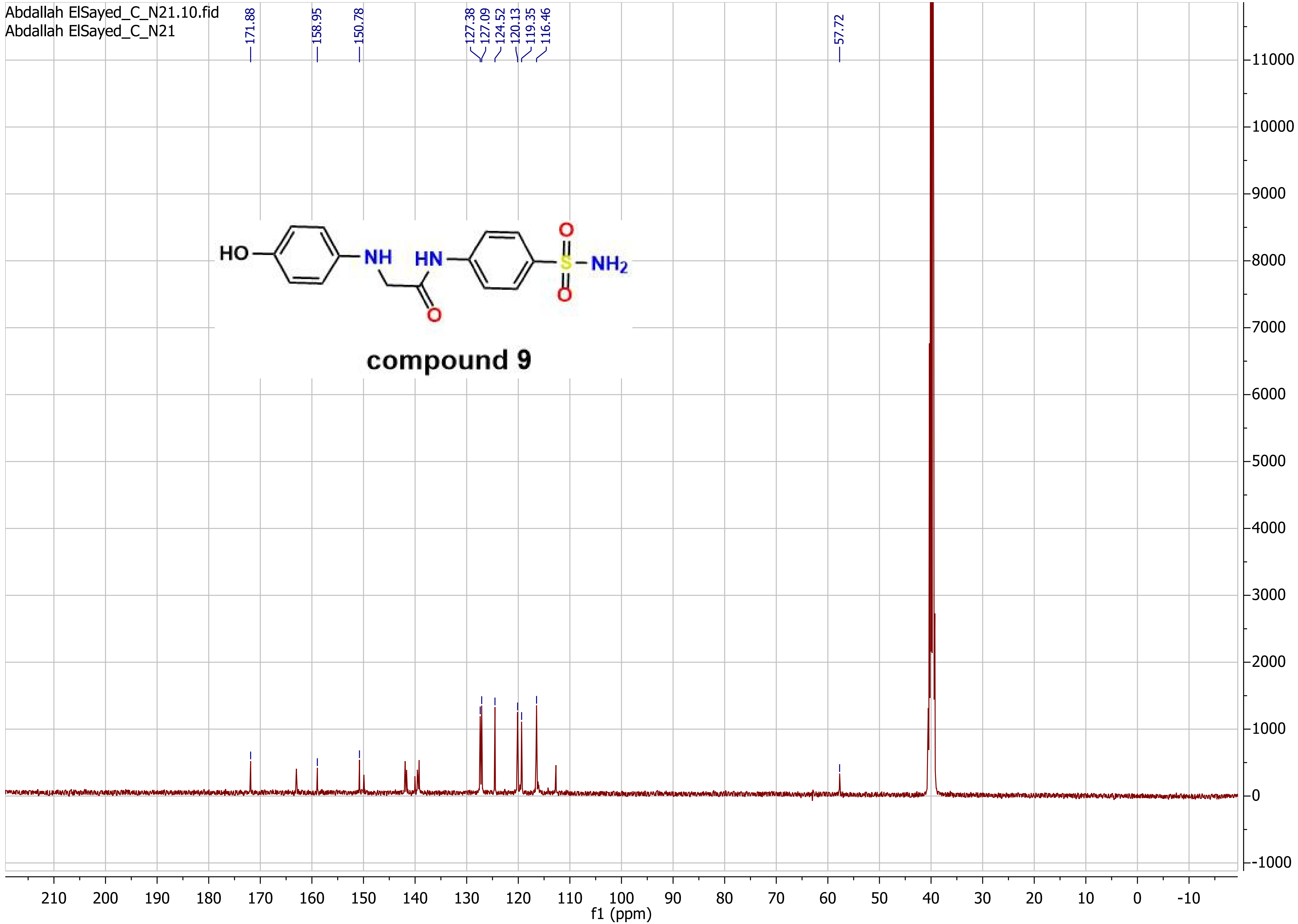
Abdallah ElSayed_C_N21.10.fid
Abdallah ElSayed_C_N21
171.88
158.95
150.78
127.38
127.09
124.52
120.13
119.35
116.46
57.72
HO
NH
HN
O
S
NH2
O
O
compound 9
f1 (ppm)

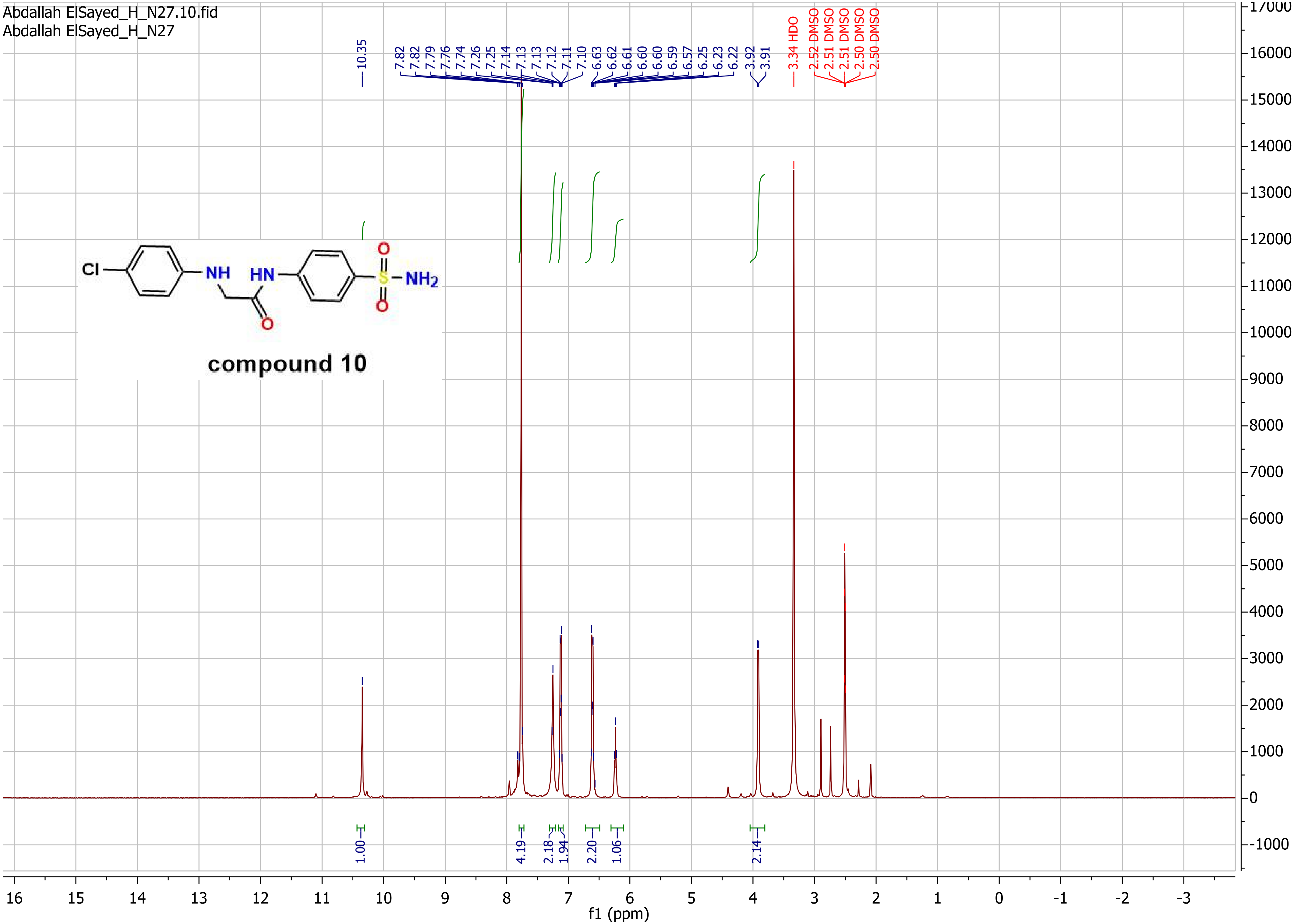
Abdallah ElSayed_H_N27.10.fid
Abdallah ElSayed_H_N27
10.35
7.82
7.82
7.79
7.76
7.74
7.26
7.25
7.14
7.13
7.13
7.12
7.11
7.10
6.63
6.62
6.61
6.60
6.60
6.59
6.57
6.25
6.23
6.22
3.92
3.91
3.34 HDO
2.52 DMSO
2.51 DMSO
2.51 DMSO
2.50 DMSO
2.50 DMSO
Cl
NH
HN
O
S
NH2
O
O
compound 10
1.00
4.19
2.18
1.94
2.20
1.06
2.14
f1 (ppm)

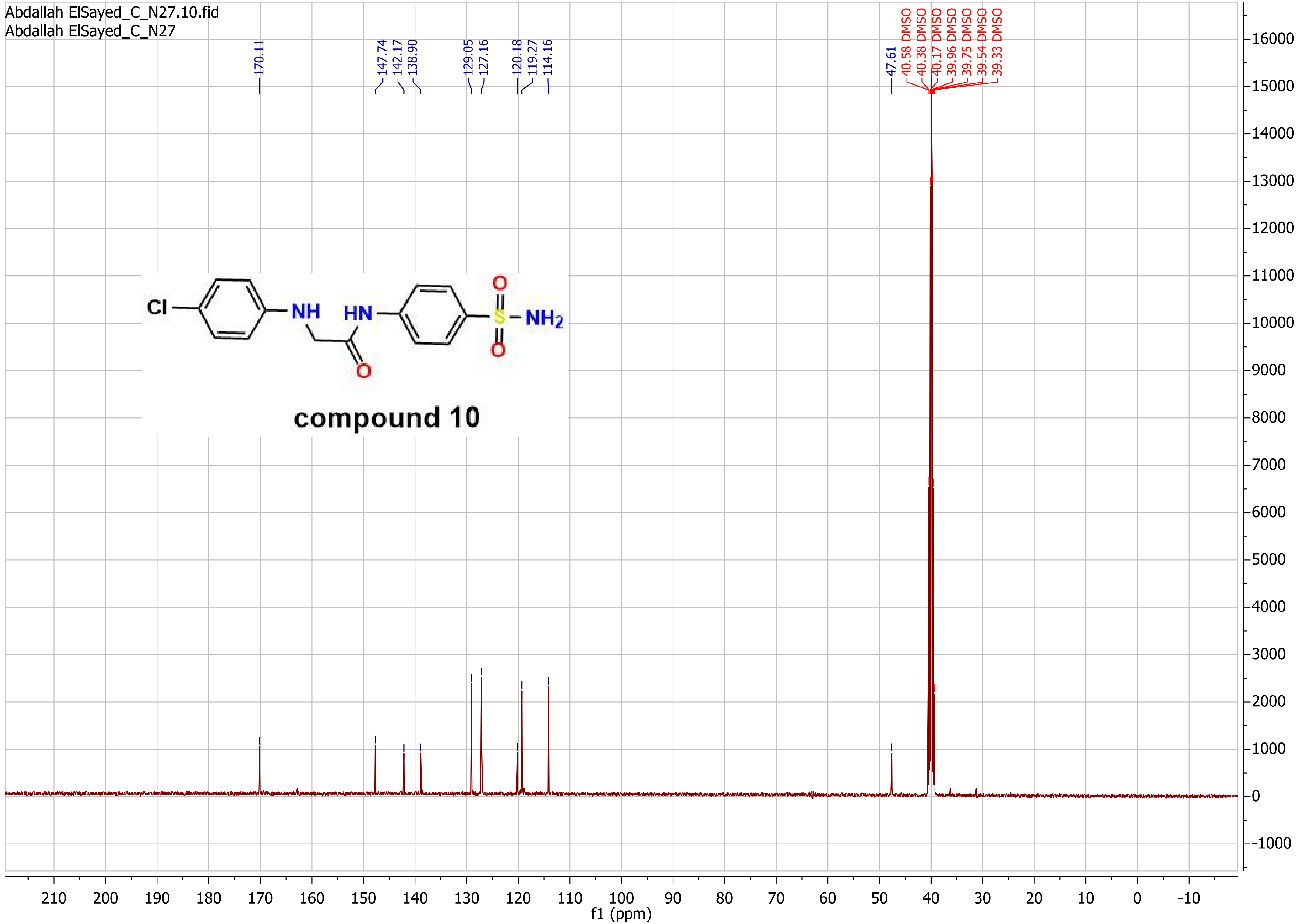

Abdallah ElSayed_C_N27.10.fid
Abdallah ElSayed_C_N27
170.11
147.74
142.17
138.90
129.05
127.16
120.18
119.27
114.16
47.61
40.58 DMSO
40.38 DMSO
40.17 DMSO
39.96 DMSO
39.75 DMSO
39.54 DMSO
39.33 DMSO
Cl
NH
HN
O
S
NH2
compound 10
f1 (ppm)

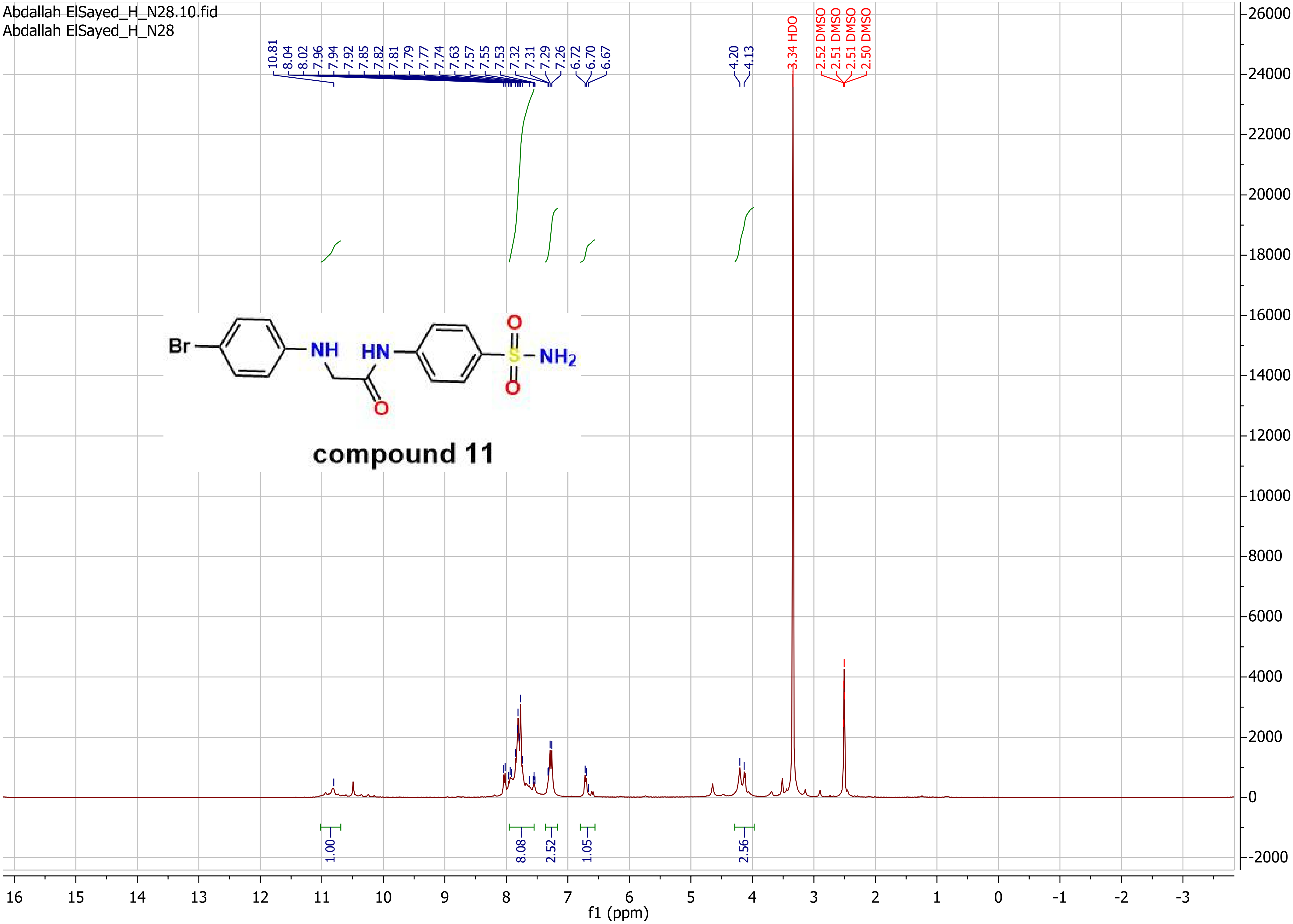

Abdallah ElSayed_H_N28.10.fid
Abdallah ElSayed_H_N28
10.81
8.04
8.02
7.96
7.94
7.92
7.85
7.82
7.81
7.79
7.77
7.74
7.63
7.57
7.55
7.53
7.32
7.31
7.29
7.26
6.72
6.70
6.67
4.20
4.13
3.34 HDO
2.52 DMSO
2.51 DMSO
2.51 DMSO
2.50 DMSO
Br
NH
HN
O
S
NH2
O
O
compound 11
1.00
8.08
2.52
1.05
2.56
f1 (ppm)

Abdallah ElSayed_C_N28.10.fid
Abdallah ElSayed_C_N28

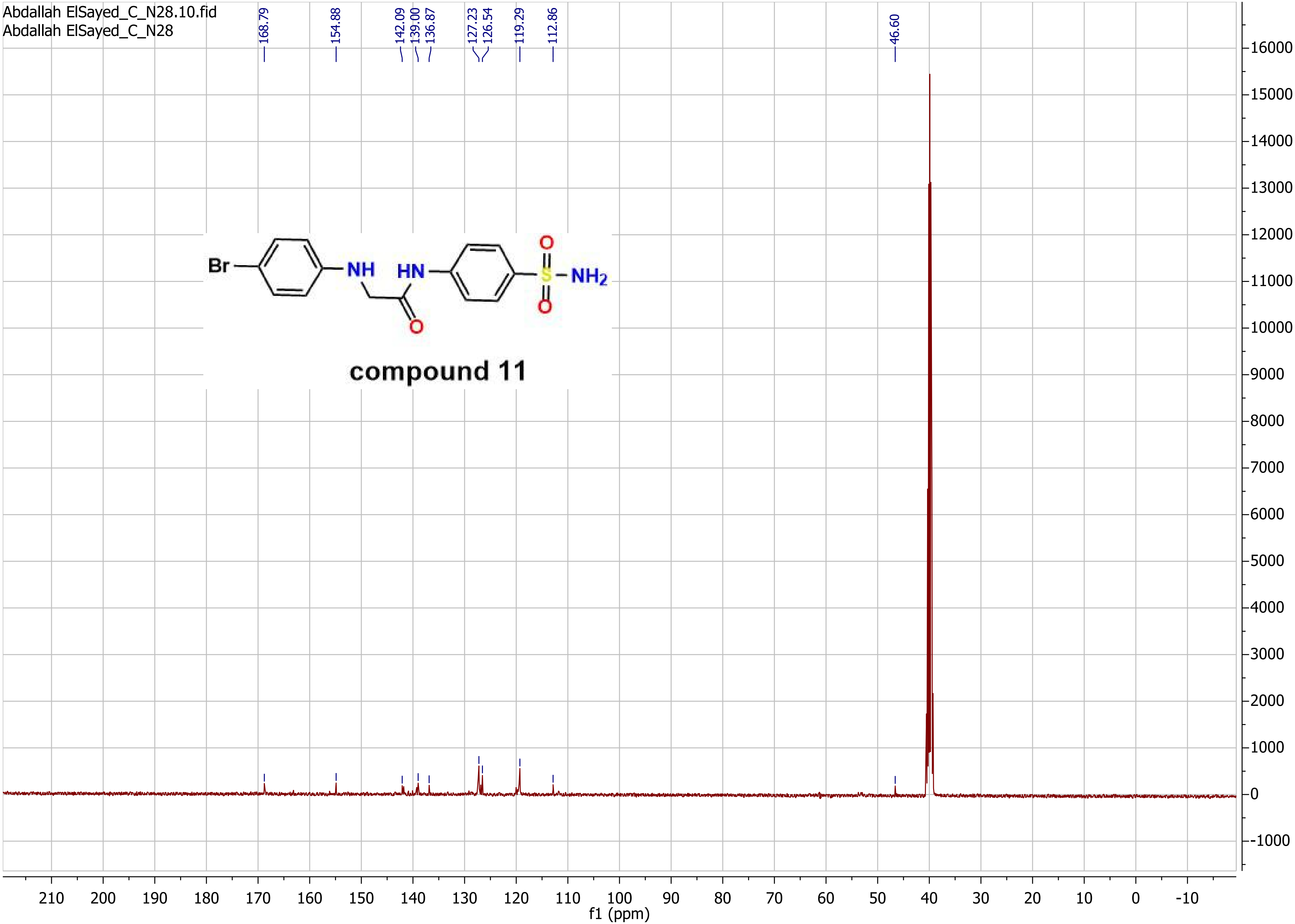

Biological part

Materials and methods

Cell Lines and Culture Conditions

The human cell lines used in this study, including normal lung fibroblasts (WI-38; ATCC CCL-75), hepatocellular carcinoma cells (HepG-2; ATCC HB-8065), lung carcinoma cells (A549; ATCC CCL-185), and breast adenocarcinoma cells (MCF-7; ATCC HTB-22), were purchased from the American Type Culture Collection (ATCC, USA) and supplied via VACSERA, Egypt. Cell line authentication was verified by ATCC using short tandem repeat (STR) analysis. The WI-38 and A549 cell lines were maintained in Dulbecco's Modified Eagle's Medium (DMEM; SERANA, Germany), while the HepG-2 and MCF-7 cell lines were cultured in RPMI-1640 medium (Lonza, USA). Both media were supplemented with 10% fetal bovine serum (FBS; SERANA, Germany) and 1% penicillin-streptomycin. All cell lines were incubated at 37°C in an atmosphere containing 5% CO_2 and 90% humidity. Prior to experimental use, all cell lines underwent routine screening to ensure the absence of mycoplasma contamination using a PCR-based detection assay.

In Vitro Cytotoxicity Assay

The cytotoxic profiles of the newly synthesized compounds (**8–11**) were evaluated using the standard 3-(4,5-dimethylthiazol-2-yl)-2,5-diphenyltetrazolium bromide (MTT) colorimetric method with slight modification [1]. 5-Fluorouracil (5-FU) was employed as the positive reference chemotherapy drug. Briefly, cells were seeded into 96-well tissue culture plates at a density of 5×10^3 cells per well and allowed to adhere overnight. The cells were subsequently exposed to varying concentrations of the test compounds (0–316.46 µM). Following a 48h incubation period, cell viability was quantified via the MTT assay. The concentrations required to inhibit 50% of cell growth (IC_{50} for cancer cells) or induce 50% cytotoxicity (CC_{50} for normal cells) were calculated using non-linear regression analysis in GraphPad Prism 8.0 software. To assess the safety margin and therapeutic potential of the tested compounds, the selectivity index (SI) was calculated as the ratio of the CC_{50} values obtained in normal WI-38 cells to the IC_{50} values obtained in the respective cancer cell lines. To examine the morphological alterations induced by the synthetic derivatives, the cancer cell lines were treated with

their IC_{50} doses for 48 h and the changes in cellular architecture, density, and apoptotic features were observed and documented using an inverted microscope (Euromex, Netherlands). All independent experiments were performed in triplicate and data were compared against untreated vehicle control cells.

Apoptosis assessment

Fluorescence Microscopy Examination

To investigate the morphological features of early and late apoptosis, single staining with propidium iodide (PI) and dual staining with acridine orange/ethidium bromide (AO/EB) were performed. The overnight incubated MCF-7 cells were treated with compound 10 at concentrations of 7, 15, and 30 µM for 48 h. Following the incubation period, the culture medium was aspirated, and the cells were washed twice with phosphate-buffered saline (PBS). The cells were then incubated with either the AO/EB dual-staining mixture or the PI single-staining (10 µg/L) solution for 15–20 min at room temperature in the dark. After staining, the cells were washed three times with PBS to remove excess dye and immediately visualized under a fluorescence microscope equipped with a digital imaging system (Olympus, USA).

Scratch method

To evaluate the migratory inhibition of the tested compound on the cancer cells, a scratch assay was performed. MCF-7 cells were seeded into sterile 12-well plates at a density of 1×10^5 cells/well and cultured until they reached approximately 90% confluence. A linear scratch was subsequently introduced into the confluent monolayer using a sterile pipette tip. After washing three times with fresh medium, medium supplemented with FBS (5%) containing compound 10 at varying concentrations (3, 7, and 15 µM) were added and incubated for 48 h. Cell migration was monitored, and images of the scratched areas were captured at 0 and 48 h using a bright-field inverted microscope (Euromex, Netherlands). The scratch areas were quantified using image analysis software, and the percentage of wound closure was calculated by relating the scratch area at 48 hours to the initial scratch area at 0 hours for each group. The experiments were performed in triplicate and compared against untreated control cells [2, 3] .

***In vitro* HDAC inhibition assay**

The inhibitory activities of compound 10 against histone deacetylases 1 and 2 (HDAC1 and HDAC2) were evaluated using commercial fluorometric HDAC1 and HDAC2 inhibitor screening kits (BPS Bioscience, USA), according to the manufacturer's instructions. Briefly, the enzyme preparations were incubated in 96-well plates with compound 10, the appropriate assay buffer, and the corresponding fluorogenic substrates at 37 °C for 30 min. The reactions were subsequently developed by adding the lysine developer, followed by incubation at room temperature for an additional 30 min. Double-distilled water (ddH_2O) was used as the vehicle control, and the corresponding enzyme activity was defined as 100%. A blank containing all assay components except the enzyme was included to determine background fluorescence. Entinostat served as the reference HDAC inhibitor. Fluorescence was measured using a microplate reader at excitation and emission wavelengths of 370 and 450 nm, respectively. After correction for background fluorescence, relative HDAC activity was calculated as the percentage of the vehicle-control activity. The percentage of HDAC inhibition was calculated as 100 minus the relative HDAC activity and all results were expressed as mean ± standard deviation (SD).

Annexin V-FITC apoptosis assay

To quantify the percentages of apoptotic and necrotic cell populations, an Annexin V-fluorescein isothiocyanate (FITC)/propidium iodide (PI) dual-staining assay was performed. MCF-7 cells were treated with the calculated IC_{50} concentration of compound 10 for 48 h. The cells were harvested, centrifuged, and washed with cold PBS and stained with Annexin V-FITC and PI solutions. The stained cells were subsequently analyzed via flow cytometry using the appropriate FITC (Annexin V) and PI emission channels to distinguish early apoptotic, late apoptotic, and necrotic cells. The acquired data were processed and quantified using FCS Express software (DeNovo Software) [4].

Gene expression evaluation

For gene expression, MCF-7 cells were treated with the IC_{50} dose of compound **10** for 48 h. Total RNA was isolated using the GeneJET Purification Kit (Thermo Scientific, USA) and reverse-transcribed. RT-qPCR was performed using SYBR Green Master Mix with specific forward/reverse primers targeting tumor suppressor (p53), oncogene (Bcl-2), and vascular endothelial growth factor (VEGF) genes. The specific forward and reverse

primer sequences utilized for gene amplification as following: 5′-TAACAGTTCCTGCATGGGCGGC-3′; 5′-AGGACAGGCACAAACACGCACC-3′ for the p53 gene, 5′-TCCGATCAGGAAGGCTAGAGTT-3′/5′-TCGGTCTCCTAAAAGCAGGC-3′ for Bcl-2; and 5'-GGCTTTACTGCTGTACCTCC-3'/ 5'-CAAATGCTTTCTCCGCTCT-3' for the VEGF gene. Relative gene expression levels were normalized against an appropriate housekeeping GAPDH gene and calculated using the comparative $2^{-\Delta\Delta Ct}$ method comparing treated and untreated MCF-7 cells.

Effect of synthetic compounds on caspase-9 and IL-6 levels

Intracellular caspase-9 and interleukin-6 (IL-6) protein levels were measured using commercial ELISA kits (ELK Biotechnology, China; Cat. Nos. ELK1530 and ELK1156, respectively), according to the manufacturers' instructions. Briefly, MCF-7 cells were treated with compound 10 at its IC_{50} concentration for 48 h, after which cell lysates were collected for analysis. The absorbance was measured at 450 nm using an ELISA microplate reader (INNO, South Korea), following the respective assay protocols.

Cell death analysis

Microscopic evaluation revealed pronounced morphological alterations in the treated A549, HepG-2, and MCF-7 cell lines compared to their respective untreated controls. Upon exposure to the IC_{50} doses of the tested compounds (**8-11**), all three cancer lines exhibited classic cytological hallmarks of apoptosis. These changes were characterized primarily by substantial cellular shrinkage, rounding, loss of characteristic monolayer density, and noticeable nuclear condensation. Taken together, these observable phenotypic deviations strongly suggest that the synthesized derivatives exert their anti-proliferative effects via the initiation of apoptosis pathways. To further elucidate the cellular death mechanisms driven by the chemical modifications, MCF-7 cells were exposed to various concentrations of the compound **10** for 48 h. Single propidium iodide (PI) staining revealed distinct, dose-dependent signs of mechanical cell death, prominently featuring chromatin condensation, nuclear shrinkage, and extensive nuclear fragmentation in treated populations. In stark contrast, untreated control cells exhibited minimal, uniformly faint background fluorescence, indicating intact nuclear architecture Complementing these findings, dual acridine orange/ethidium bromide (AO/EB) staining provided clear visual confirmation of a concentration-dependent transition through the

apoptotic cascade. While healthy vehicle control cells emitted a uniform green fluorescence signifying normal membrane integrity and viable cells, treated MCF-7 cells exhibited a distinct shift in color. At lower exposure limits, the appearance of yellow and greenish-orange punctate fluorescence indicated early and late apoptotic populations actively shifting under chemical stress. At the high dose, the cell population progressed entirely to a dense, bright orange-to-reddish fluorescence profile, signaling highly compromised cell membranes characteristic of end-stage apoptotic populations.

Statistical Analysis

All experiments were performed in triplicate, and results are expressed as mean ± SEM. Statistical analysis was conducted using GraphPad Prism software (version 8.0). Differences between groups were analyzed using one-way analysis of variance (ANOVA), with statistical significance considered at $p < 0.05$.

1. Dawoud, N.T., et al., *Synthesis, and docking studies of novel heterocycles incorporating the indazolylthiazole moiety as antimicrobial and anticancer agents.* Scientific Reports, 2022. **12**(1): p. 3424.
2. Ebraheem, M.A., et al., *Purification and characterization of the produced hyaluronidase by Brucella Intermedia MEFS for antioxidant and anticancer applications.* Microbial Cell Factories, 2024. **23**(1): p. 200.
3. El-Didamony, S.E., et al., *Correction: Melittin alcalase-hydrolysate: a novel chemically characterized multifunctional bioagent; antibacterial, anti-biofilm and anticancer.* Frontiers in Microbiology, 2026. **17**: p. 1988804.
4. Serag, E., et al., *Metal–organic framework MIL-101 (Fe) functionalized with folic acid as a multifunctional nanocarrier for targeted chemotherapy–photodynamic therapy.* Biomaterials Science, 2025. **13**(9): p. 2351–2367.

6042 Cornerstone Court W, Ste B
San Diego, CA 92121
Tel: 1.858.202.1401
Fax: 1.858.481.8694
Email: info@bpsbioscience.com

Data Sheet

Fluorogenic HDAC1 Assay Kit
Catalog #: 50061

DESCRIPTION: The *Fluorogenic HDAC1 Assay Kit* is a complete assay system designed to measure histone deacetylase 1 (HDAC1) activity for screening and profiling applications. It comes in a convenient 96-well format, with all the reagents necessary for 100 fluorescent HDAC1 activity measurements. In addition, the kit includes purified HDAC1 enzyme and a potent HDAC inhibitor, Trichostatin A, for use as a positive and negative control. The *Fluorogenic HDAC1 Assay Kit* is based on a unique fluorogenic substrate and developer combination. This assay method eliminates dealing with the radioactivity, extraction, and chromatography aspects of traditional assays. Using this kit, only two simple steps on a microtiter plate are needed to analyze the HDAC activity level. First, the HDAC fluorometric substrate, containing an acetylated lysine side chain, is incubated with purified HDAC1. The deacetylation sensitizes the substrate so subsequent treatment with the Lysine Developer produces a fluorophore that can then be measured using a fluorescence reader.

HDACs regulate cellular processes by catalyzing the hydrolysis of an acetyl group from acetyllysines in modified proteins. In the HDAC assay, fluorescent-dye molecules are attached to a peptide containing acetyllysine. Attachment to the peptide quenches the fluorescence of the dye. After treatment of the peptide with an HDAC, the reaction is mixed with a development solution that is specific for nonacetylated lysines. If the acetyl group has been removed from the lysine by the HDAC, this solution will release the dye allowing for fluorescence. Fluorescence is therefore directly related to HDAC activity.

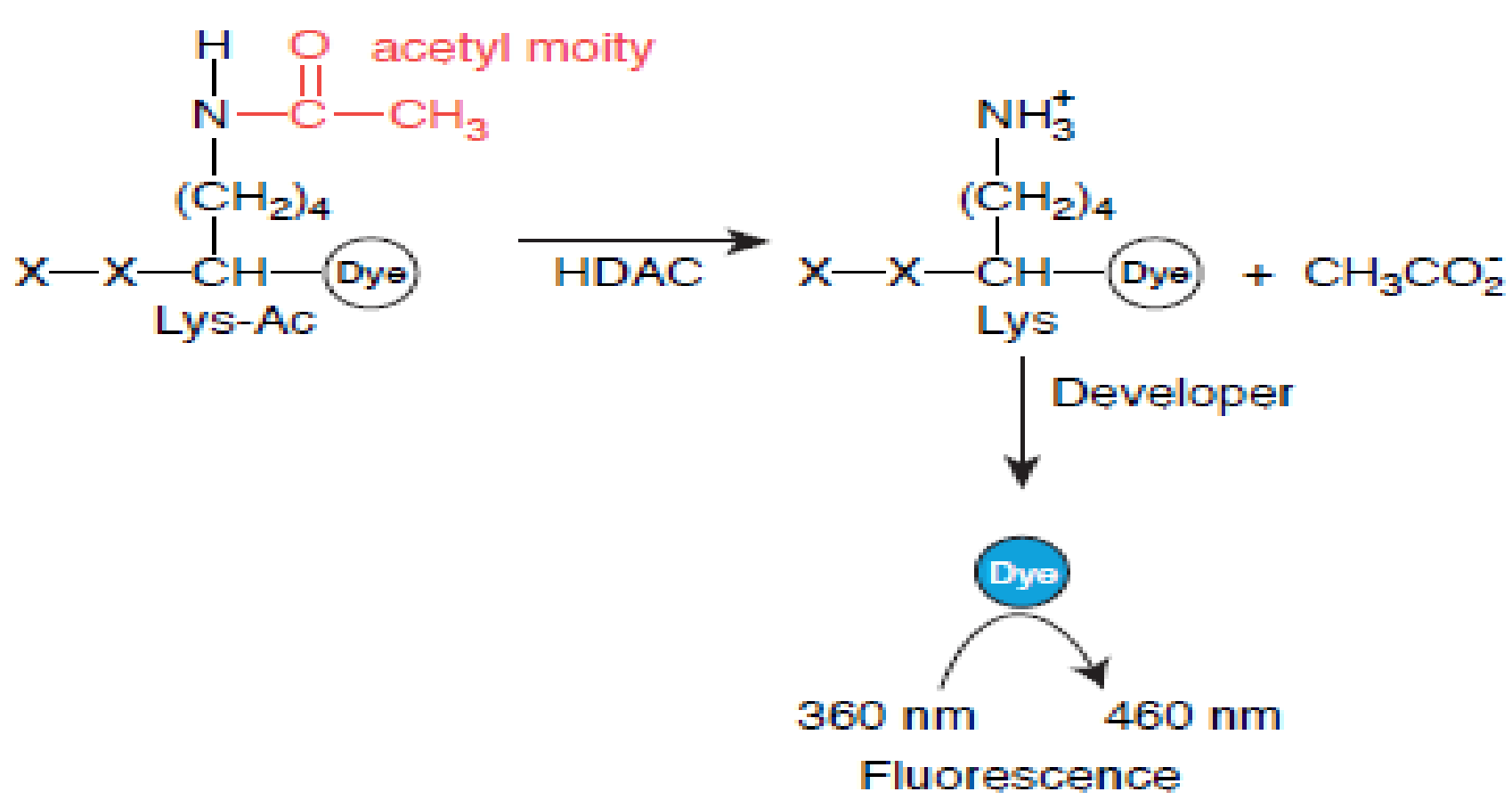

OUR PRODUCTS ARE FOR RESEARCH USE ONLY. NOT FOR DIAGNOSTIC OR THERAPEUTIC USE.
To place your order, please contact us by Phone **1.858.202.1401** Fax **1.858.481.8694**
Or you can Email us at: **info@bpsbioscience.com**
Please visit our website at: **www.bpsbioscience.com**

6042 Cornerstone Court W, Ste B
San Diego, CA 92121
Tel: 1.858.202.1401
Fax: 1.858.481.8694
Email: info@bpsbioscience.com

COMPONENTS:

Catalog #	Reagent	Amount	Storage	
50051	HDAC1 human recombinant enzyme	1 µg	-80°C	***Avoid Freeze/ Thaw Cycles!***
50037	Fluorogenic HDAC substrate 3 (5 mM)	50 µl	-80°C	
50030	2x HDAC Developer (contains Trichostatin A) (50 µM)	6 ml	-80°C	
	Trichostatin A (200 µM) in DMSO	100 µl	-20°C	
50031	HDAC Assay Buffer	10 ml	-20°C	
79685	black, low binding NUNC black microtiter plate	1 plate	Room temp.	

MATERIALS OR INSTRUMENTS REQUIRED BUT NOT SUPPLIED:
0.1% solution (1 mg/ml) of bovine serum albumin (BSA) in water
Fluorimeter capable of excitation at 350-380 nm and detection at 440-460 nm
Adjustable micropipettor and sterile tips
Rotating or rocker platform

APPLICATIONS: Great for studying enzyme kinetics and screening small molecular inhibitors for drug discovery and HTS applications.

STABILITY: One year from date of receipt when stored as directed.

REFERENCE(S):

1. Santo, L., *et al., Blood.* 2012 Mar 15;**119(11)**:2579-89.
2. Bradner, J.E., *et al., Nat Chem Biol.* 2010 Mar;**6(3)**: 238-243.

ASSAY PROTOCOL:

Immediately prior to assay:

1) Dilute **Trichostatin A (200 µM)** stock 10-fold with **HDAC Assay Buffer** to make a 20 µM solution. (Make only sufficient quantity needed for the assay; store remaining 200 µM **Trichostatin A** stock solution in aliquots at -80°C).

2) Dilute **HDAC substrate 3 (5 mM)** stock 25-fold with **HDAC Assay Buffer** to make a 200 µM solution. (Make only sufficient quantity needed for the assay; store remaining 5 mM stock solution in aliquots at -80°C).

3) Dilute **HDAC1** in **HDAC Assay Buffer** to 1.4 ng/µl (7 ng/reaction)*. Aliquot any remaining enzyme and store undiluted at -80°C. Keep diluted enzyme on ice. Discard any remaining diluted enzyme after use. **Note: optimal enzyme concentration may vary with the specific activity of the enzyme.*

OUR PRODUCTS ARE FOR RESEARCH USE ONLY. NOT FOR DIAGNOSTIC OR THERAPEUTIC USE.
To place your order, please contact us by Phone **1.858.202.1401** Fax **1.858.481.8694**
Or you can Email us at: **info@bpsbioscience.com**
Please visit our website at: **www.bpsbioscience.com**

6042 Cornerstone Court W, Ste B
San Diego, CA 92121
Tel: 1.858.202.1401
Fax: 1.858.481.8694
Email: info@bpsbioscience.com

Step 1:
In duplicate, add the reaction mixtures (below) to the microtiter black plate as follows:

1) Prepare the master mixture: N wells × (5 µl **HDAC substrate 3 (200 µM)** + 5 µl BSA (1 mg/ml) + 30 µl **HDAC Assay Buffer**). Add 40 µl of master mixture to all wells.

2) Add 5 µl of inhibitor solution of each well designated "Test Inhibitor." For the "Positive Control" and "Blank," add 5 µl of 10% DMSO in water (inhibitor buffer). Add 5 µl of diluted **Trichostatin A** (20 µM) to the well designated "Inhibitor Control." *Keep final DMSO concentration at or below 1%.*

3) Add 5 µl of **HDAC Assay Buffer** to the wells designated "Blank."

4) Initiate reaction by adding 5 µl of diluted **HDAC1** enzyme to the wells designated "Positive Control," "Test Inhibitor," and "Inhibitor Control." Incubate at 37ºC for 30 min.

	**"Blank"**	**Positive Control**	**Test Inhibitor**	**Inhibitor Control**
HDAC substrate 3 (200 µM)	5 µl	5 µl	5 µl	5 µl
BSA (1 mg/ml)	5 µl	5 µl	5 µl	5 µl
HDAC Assay Buffer	35 µl	30 µl	30 µl	30 µl
Diluted Trichostatin A (20 µM)	–	–	–	5 µl
Test Inhibitor	–	–	5 µl	–
10% DMSO in water (Inhibitor buffer)	5 µl	5 µl	–	
Diluted HDAC1 (1.4 ng/µl)	–	5 µl	5 µl	5 µl
Total	50 µl	50 µl	50 µl	50 µl

Step 2:
Add 50 µl of undiluted **HDAC Developer (2x)** to each well. Incubate the plate at room temperature for 15 minutes.

Step 3:
Read sample in a microtiter plate-reading fluorimeter capable of excitation at a wavelength in the range of 350-380 nm and detection of emitted light in the range of 440-460 nm. "Blank" value is subtracted from all other values.

OUR PRODUCTS ARE FOR RESEARCH USE ONLY. NOT FOR DIAGNOSTIC OR THERAPEUTIC USE.
To place your order, please contact us by Phone **1.858.202.1401** Fax **1.858.481.8694**
Or you can Email us at: **info@bpsbioscience.com**
Please visit our website at: **www.bpsbioscience.com**

6042 Cornerstone Court W, Ste B
San Diego, CA 92121
Tel: 1.858.202.1401
Fax: 1.858.481.8694
Email: info@bpsbioscience.com

Example of Assay Results:

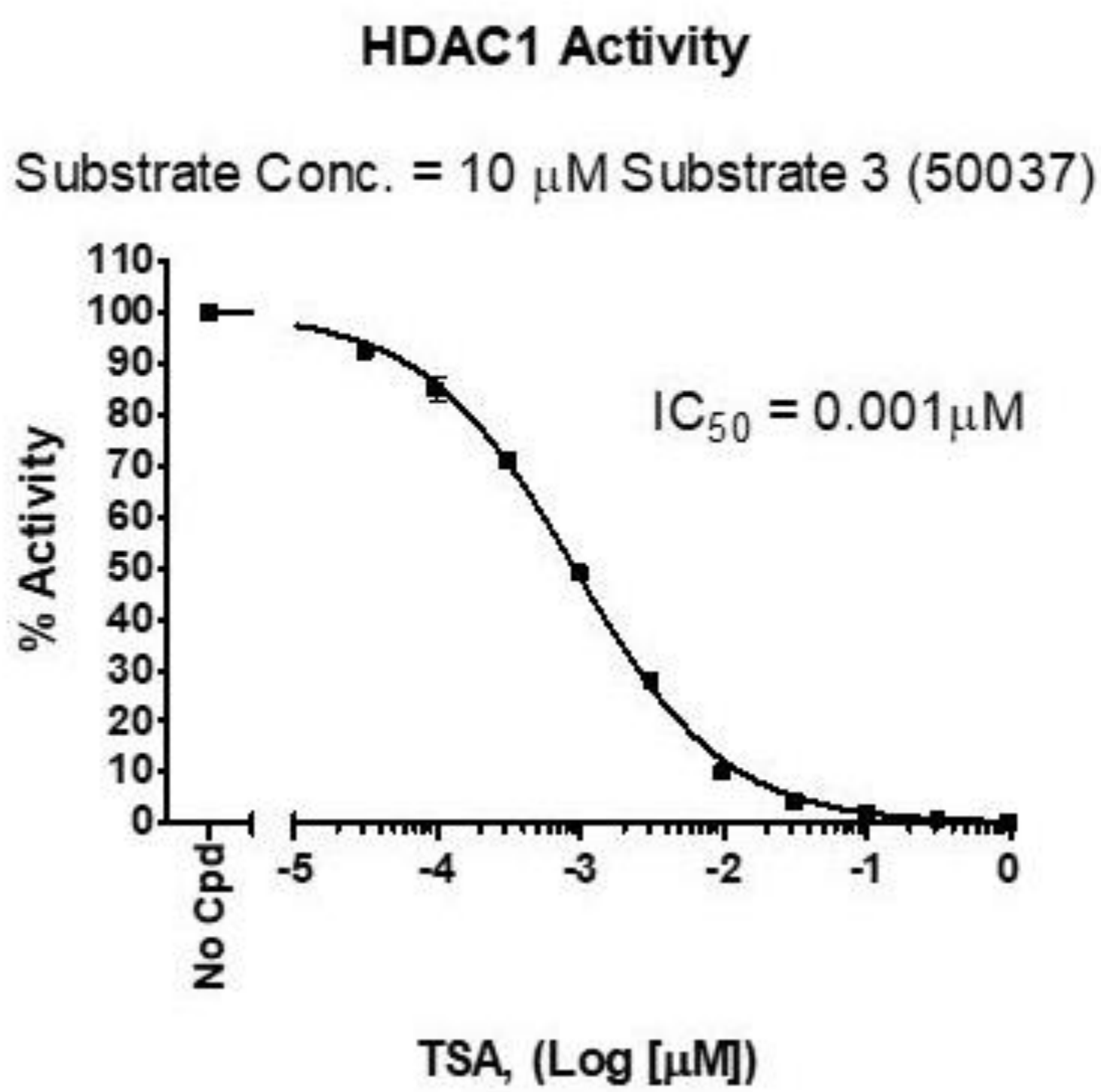

HDAC1 enzyme activity, measured using the *HDAC1 Fluorogenic Assay Kit*, BPS Bioscience Catalog #50061. Fluorescence was measured using a Bio-Tek fluorescent microplate reader. *Data shown is lot-specific. For lot-specific information, please contact BPS Bioscience, Inc. at info@bpsbioscience.com.*

6042 Cornerstone Court W, Ste B
San Diego, CA 92121
Tel: 1.858.202.1401
Fax: 1.858.481.8694
Email: info@bpsbioscience.com

RELATED PRODUCTS:

Product Name	Catalog #	Size
HDAC1	50051	50 µg
HDAC2 (C-His)	50002	50 µg
HDAC2 (C-Flag)	50052	50 µg
HDAC3/NcoR2	50003	50 µg
HDAC4	50004	10 µg
HDAC5	50005	10 µg
HDAC6 (C-Flag)	50056	50 µg
HDAC6 (N-GST)	50006	50 µg
HDAC6 (H216A)	50046	50 µg
HDAC6 (H611A)	50066	50 µg
HDAC7	50007	10 µg
HDAC8	50008	50 µg
HDAC9	50009	10 µg
HDAC10	50010	50 µg
HDAC11	50011	50 µg
HDAC Assay Kit	50033	96 reactions
HDAC Assay Kit (Green)	50034	96 reactions
HDAC Class 2a Assay Kit	50041	96 reactions
HDAC2 Assay Kit	50062	96 reactions
HDAC3 Assay Kit	50073	96 reactions
HDAC6 Assay Kit	50076	96 reactions
HDAC8 Assay Kit	50068	96 reactions

6044 Cornerstone Court W, Ste E
San Diego, CA 921211
Tel: 1.858.202.1401
Fax: 1.858.481.8694
Email: info@bpsbioscience.com

Data Sheet

Kinetic HDAC2 Assay Kit

Catalog #: 53002

DESCRIPTION: The *Kinetic HDAC2 Assay Kit* is a complete assay system designed to measure histone deacetylase 2 (HDAC2) activity kinetically for screening and profiling applications. It comes in a convenient 96-well format, with all the reagents necessary for 100 fluorescent HDAC2 activity measurements. In addition, the kit includes purified HDAC2 enzyme and a potent HDAC inhibitor, SAHA, for use as a positive and negative control. The *Kinetic HDAC2 Assay Kit* is based on a unique fluorogenic substrate and developer combination. This assay method eliminates dealing with the radioactivity, extraction, and chromatography aspects of traditional assays. Using this kit, only one simple step for setting up the HDAC2 reaction on a microtiter plate is needed and then the HDAC2 activity level can be measured kinetically using a fluorescence reader.

COMPONENTS:

<table>
<tr><th>Catalog #</th><th>Reagent</th><th>Amount</th><th colspan="2">Storage</th></tr>
<tr><td>50002</td><td>HDAC2 human recombinant enzyme</td><td>5 µg</td><td>-80°C</td><td rowspan="8">Avoid Freeze/ Thaw Cycles!</td></tr>
<tr><td>50037</td><td>Fluorogenic HDAC substrate 3 (5 mM)</td><td>50 µl</td><td>-80°C</td></tr>
<tr><td>53029</td><td>10x HDAC Developer</td><td>1 ml</td><td>-80°C</td></tr>
<tr><td></td><td>Developer Dilution Buffer</td><td>10 ml</td><td>-20°C</td></tr>
<tr><td></td><td>SAHA (1 mM)</td><td>20 µl</td><td>-20°C</td></tr>
<tr><td>50031</td><td>HDAC Assay Buffer</td><td>20 ml</td><td>-20°C</td></tr>
<tr><td></td><td>BSA (1mg/ml)</td><td>1 ml</td><td>-20°C</td></tr>
<tr><td>79685</td><td>black, low binding NUNC black microtiter plate</td><td>1 plate</td><td>Room temp.</td></tr>
</table>

MATERIALS OR INSTRUMENTS REQUIRED BUT NOT SUPPLIED:
Fluorescent microplate reader

APPLICATIONS: Great for studying enzyme kinetics and screening small molecular inhibitors for drug discovery and HTS applications.

STABILITY: One year from date of receipt when stored as directed.

REFERENCE(S):

1. Santo, L., *et al., Blood.* 2012 Mar 15;**119(11)**:2579-89.
2. Bradner, J.E., *et al., Nat Chem Biol.* 2010 Mar;**6(3)**: 238-243.

6044 Cornerstone Court W, Ste E
San Diego, CA 921211
Tel: 1.858.202.1401
Fax: 1.858.481.8694
Email: info@bpsbioscience.com

ASSAY PROTOCOL:

Thaw all of the components except the HDAC enzyme, and place on ice. All of the reactions should be set up on ice.

1) Prepare the master **HDAC Substrate Solution**
   To make the master **HDAC Substrate Solution,** mix 2368 µl **HDAC Assay Buffer,** 800 µl BSA (1mg/ml), and 32 µl **HDAC Substrate.** This is enough for 100 reactions. If you have less than 100 reactions, you can reduce the components proportionally. (Make sufficient quantity needed for the assay; store remaining BSA and 5 mM stock solution in aliquots at -80°C.)

   Add 20 µl of the master **HDAC Substrate Solution** to each well.

2) Prepare **Inhibitor Solutions**
   Dilute the Test Inhibitor 10-fold higher than the final concentration you want to test in 10% DMSO (the final DMSO concentration is 1% in all of the reactions).

   Dilute the same buffer without the inhibitor (Inhibitor Buffer) in 10% DMSO as a control.

   Add 10 µl of the **Test Inhibitor Solution** to each well designated as "Test Inhibitor", and 10 µl of **SAHA Solution** to each well designated as "SAHA Inhibitor Control". Add 10 µl of 10% DMSO to each well designated "Blank" and "Positive Control".

3) Prepare **Developer solution**
   Dilute the **10X Developer Solution** (1:10 dilution) in **Developer Dilution Buffer.**

   Add 50 µl of the **Developer Solution** to each well.

4) Prepare **HDAC2 Enzyme Solution**
   Dilute **HDAC2** in HDAC assay buffer to 2 ng/µl (40 ng/reaction). Aliquot any remaining enzyme and store undiluted at -80°C.

   Add 20 µl of HDAC assay buffer to each well designed "Blank". Add 20 µl of the **HDAC1 enzyme solution** to each well designed "Positive Control", "Test Inhibitor" and "SAHA Inhibitor Control". **Always add the enzyme solution last.**

5) Measure the plate in 5 minute intervals for a period of up to one hour, using a microtiter plate-reading fluorimeter capable of excitation in the range of 350-380

6044 Cornerstone Court W, Ste E
San Diego, CA 921211
Tel: 1.858.202.1401
Fax: 1.858.481.8694
Email: info@bpsbioscience.com

nm and detection of emitted light in the range of 440-460 nm. The "Blank" value is subtracted from all other values.

	"Blank"	Positive Control	Test Inhibitor	SAHA Inhibitor Control
Master HDAC Substrate Solution	20 µl	20 µl	20 µl	20 µl
SAHA	–	–	–	10 µl
Test Inhibitor	–	–	10 µl	–
Inhibitor buffer (no inhibitor)	10 µl	10 µl	–	
1X Developer	50 µl	50 µl	50 µl	50 µl
HDAC assay buffer	20 µl	–	–	–
HDAC2 (2 ng/µl)	–	20 µl	20 µl	20 µl
Total	100 µl	100 µl	100 µl	100 µl

Example of Assay Results:

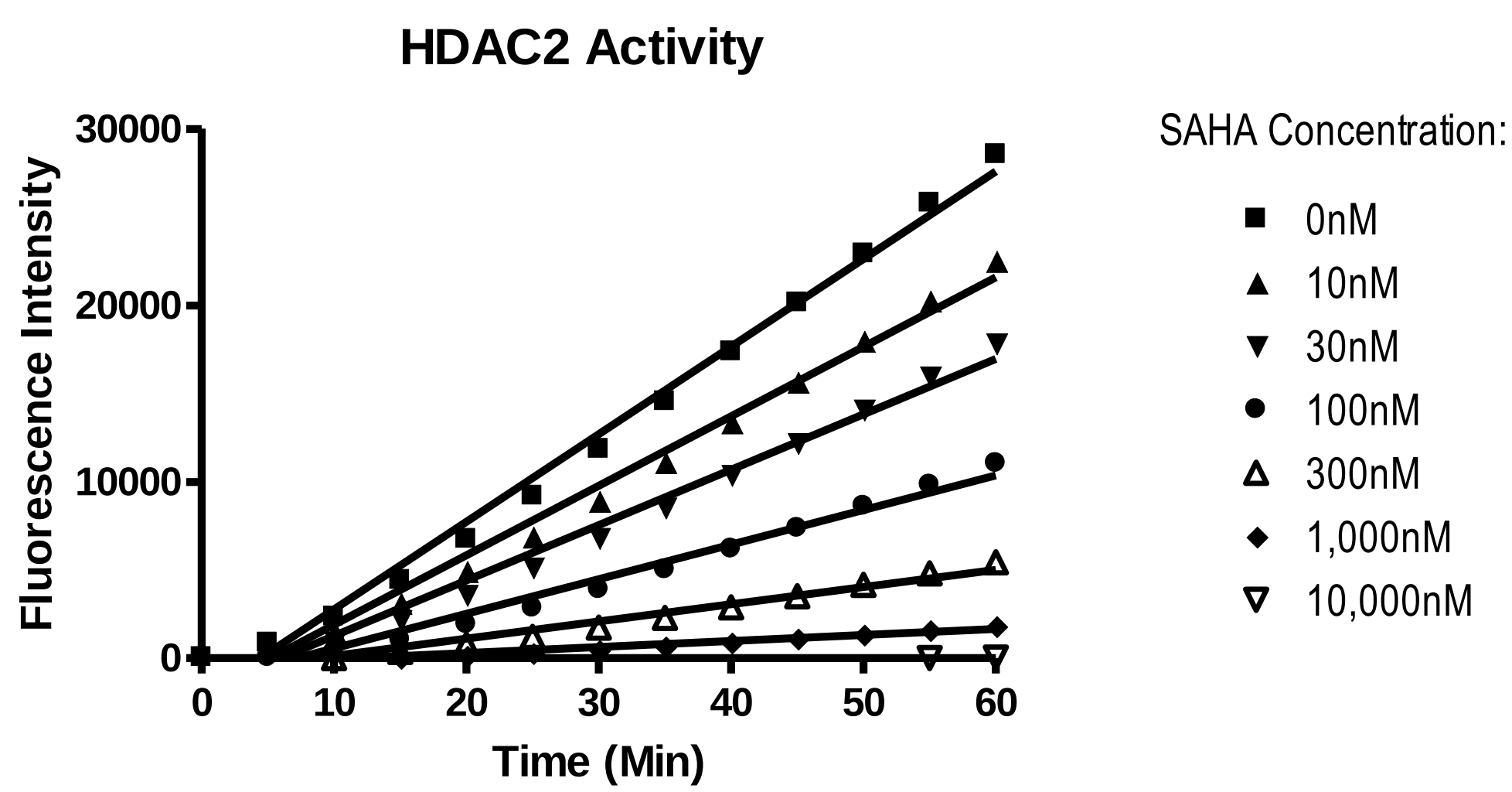

Figure 1. Time course of HDAC2 activity against different concentrations of SAHA, measured using the *Kinetic HDAC2 Assay Kit*, BPS Bioscience Cat# 53002. Fluorescence was measured using a Tecan fluorescent microplate reader. *Data shown is lot-specific. For lot-specific information, please contact BPS Bioscience, Inc. at info@bpsbioscience.com.*

6044 Cornerstone Court W, Ste E
San Diego, CA 921211
Tel: 1.858.202.1401
Fax: 1.858.481.8694
Email: info@bpsbioscience.com

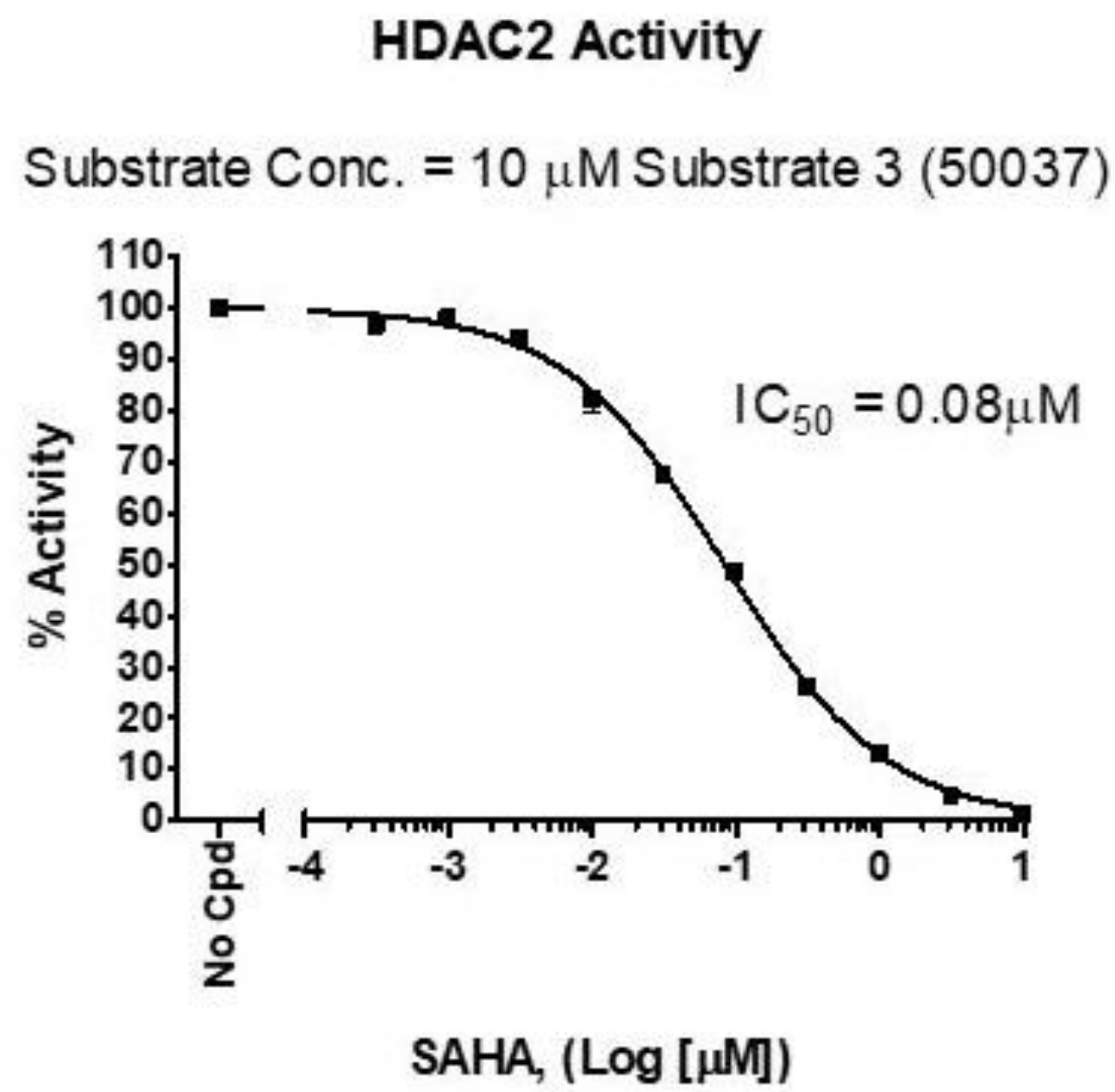

Figure 2. IC50 assay of HDAC2 against SAHA.

6044 Cornerstone Court W, Ste E
San Diego, CA 921211
Tel: 1.858.202.1401
Fax: 1.858.481.8694
Email: info@bpsbioscience.com

RELATED PRODUCTS:

Product Name	Catalog #	Size
HDAC1	50051	50 µg
HDAC2 (C-Flag)	50052	50 µg
HDAC3/NcoR2	50003	50 µg
HDAC4	50004	10 µg
HDAC5	50005	10 µg
HDAC6 (C-Flag)	50056	50 µg
HDAC7	50007	10 µg
HDAC8	50008	50 µg
HDAC9	50009	10 µg
HDAC10	50010	50 µg
HDAC11	50011	50 µg
HDAC Assay Kit	50033	96 reactions
HDAC Assay Kit (Green)	50034	96 reactions
HDAC Class 2a Assay Kit	50041	96 reactions
HDAC1 Assay Kit	50061	96 reactions
HDAC2 Assay Kit	50062	96 reactions
HDAC3 Assay Kit	50073	96 reactions
HDAC4 Assay Kit	50064	96 reactions
HDAC5 Assay Kit	50065	96 reactions
HDAC6 Assay Kit	50076	96 reactions
HDAC7 Assay Kit	50067	96 reactions
HDAC8 Assay Kit	50068	96 reactions
HDAC9 Assay Kit	50069	96 reactions

Researcher : Dr.Ismail elfakharani
email: dresmail1981@gmail.com **mob.** 01009188422
esmailelfakharany@yahoo.co.uk

Date : 28-03-2026
Assay : HDAC1 assay
Samples : 02 samples .
Reference : ---
Cell lines : --
Kit used : --

Lab Report

ser	compound		HDAC1
	code	M.W	IC50 (uM)
1	**Compound 10**	---	5.66 ± 0.19
2	**Entinostat**	---	1.04 ± 0.04

Detailed Results :

HDAC1 [redacted]

code	IC50 (uM)	Conc (µM)	log	%inh	ΔT	ΔRFU	slope	K.Activity	EC			
**Compound 10**		29.44	1	77.01	30	9231.8	1338.8	27.58223	120	9113	9392	9191
		2.944	0	36.54	30	25488.9	1338.8	76.15455	120	25161	25930	25376
		0.294	-1	13.04	30	34925.3	1338.8	104.3482	120	34476	35530	34770
		0.0294	-2	4.352	30	38416.2	1338.8	114.7781	120	37922	39081	38245
		0.0029	-3	1.484	30	39568.1	1338.8	118.2195	120	39059	40253	39392
EC				0	30	40162.7	1338.8	120	120	39646	40858	39984
	[redacted]		[redacted]		[redacted]			[redacted]				

code	IC50 (uM)	Conc (µM)	log	%inh	ΔT	ΔRFU	slope	K.Activity	EC			
Entinostat		25.85	1	85.26	30	5919.2	1338.8	17.68495	120	5843	6022	5893
		2.585	0	54.11	30	18430.1	1338.8	55.06458	120	18193	18749	18348
		0.258	-1	32.02	30	27303.3	1338.8	81.57536	120	26952	27776	27182
		0.0258	-2	15.53	30	33928.5	1338.8	101.3699	120	33492	34516	33778
		0.0025	-3	2.664	30	39094.0	1338.8	116.803	120	38591	39771	38920
EC				0	30	40162.7	1338.8	120	120	39646	40858	39984
	[redacted]		[redacted]		[redacted]			[redacted]				

Researcher : Dr.Esmail El-Fakharany
email: dresmail1981@gmail.com **mob.** 01009188422
esmailelfakharany@yahoo.co.uk
Date : 28-03-2026
Assay : HDAC2 assay
Samples : 02 compounds.
Cell line : ---
Reference : ---
Kit used : ---
Reader : TEACAN spark READER

Lab Report

ser	Compound		HDAC 2
	code	MW (g/mol)	IC50 (uM)
1	Compound 10	---	2.27 ± 0.07
***	Entinostat	---	1.21 ± 0.04

Detailed results

HDAC2

code	IC50	Conc (uM)	log	%inh	ΔT	ΔRFU	slope	K.Activity	EC			
Compound 10		29.44	1.469	83.74	30	7335.4	1503.8	19.51157	120	7241	7462	7303
		2.944	0.469	56.04	30	19831.1	1503.8	52.7494	120	19576	20174	19743
		0.294	-0.53	7.809	30	41591.1	1503.8	110.6293	120	41056	42311	41406
		0.0294	-1.53	5.314	30	42716.6	1503.8	113.623	120	42167	43456	42527
		0.0029	-2.53	2.723	30	43885.6	1503.8	116.7326	120	43321	44645	43691
EC				0	30	45113.4	1503.8	120	120	44533	45894	44913

code	IC50 (uM)	conc	log	%inh	ΔT	ΔRFU	slope	K.Activity	EC			
Entinostat		25.85	1.412	86.92	30	5901.9	1503.8	15.69871	120	5826	6004	5876
		2.585	0.412	52.76	30	21313.2	1503.8	56.6916	120	21039	21682	21218
		0.258	-0.58	23.94	30	34313.5	1503.8	91.27134	120	33872	34907	34161
		0.0258	-1.58	14.77	30	38448.7	1503.8	102.2707	120	37954	39114	38278
		0.0025	-2.58	5.294	30	42725.7	1503.8	113.6473	120	42176	43465	42536
EC				0	30	45113.4	1503.8	120	120	44533	45894	44913

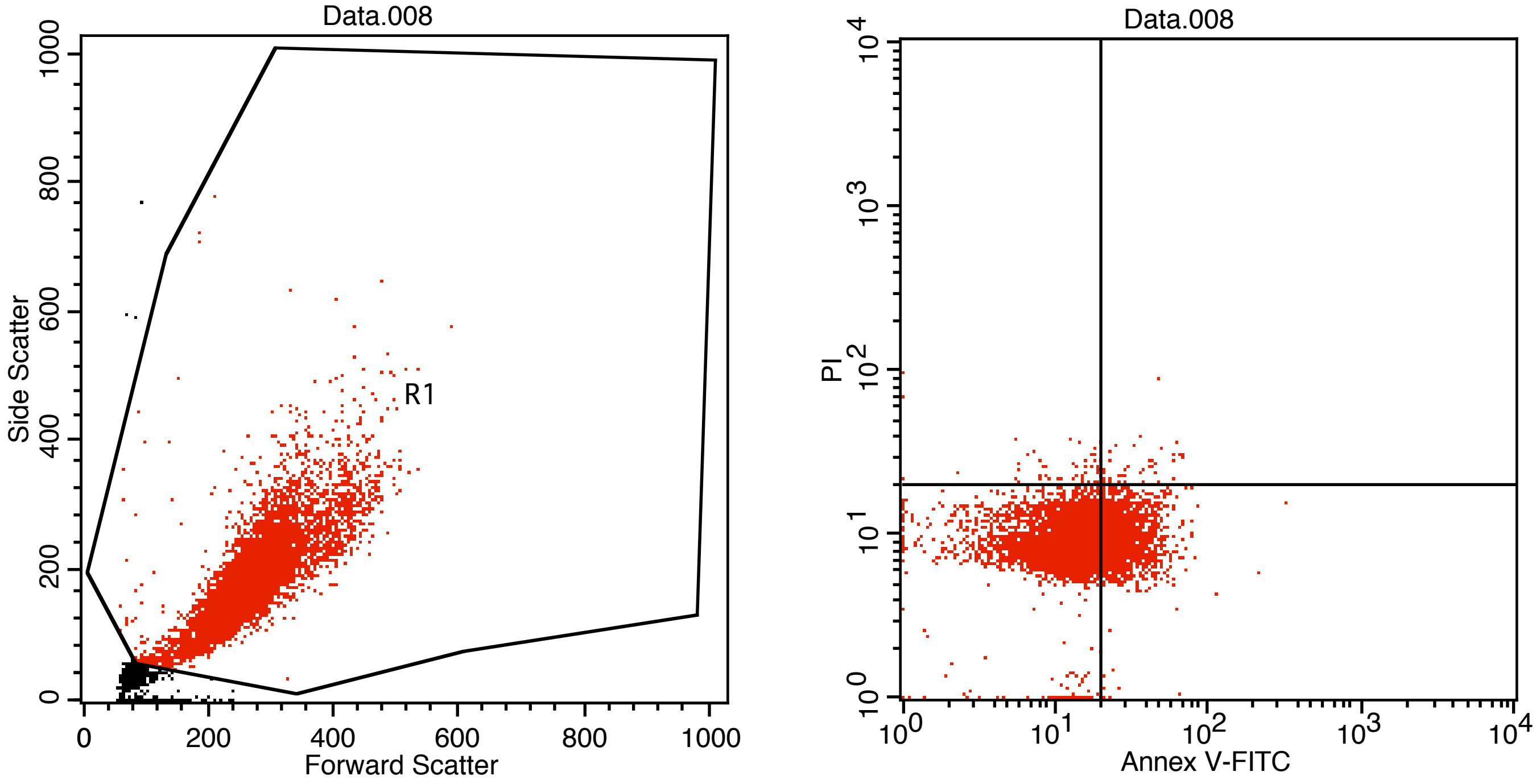

Quadrant Statistics

File: Data.008
Sample ID: CaCo2 - 27
Tube: Untitled
Acquisition Date: 03-Mar-26
Gated Events: 9529
X Parameter: Annex V-FITC (Log)
Quad Location: 20, 20

Log Data Units: Linear Values
Patient ID: Apoptosis
Panel: Untitled Acquisition Tube List
Gate: G1
Total Events: 10000
Y Parameter: PI (Log)

Quad	Events	% Gated	% Total	X Mean	X Geo Mean	Y Mean	Y Geo Mean
UL	52	0.55	0.52	12.52	10.44	26.98	25.60
UR	47	0.49	0.47	35.20	32.66	26.78	25.65
LL	7231	75.88	72.31	12.99	11.96	8.83	8.40
LR	2199	23.08	21.99	27.85	26.62	9.66	9.22

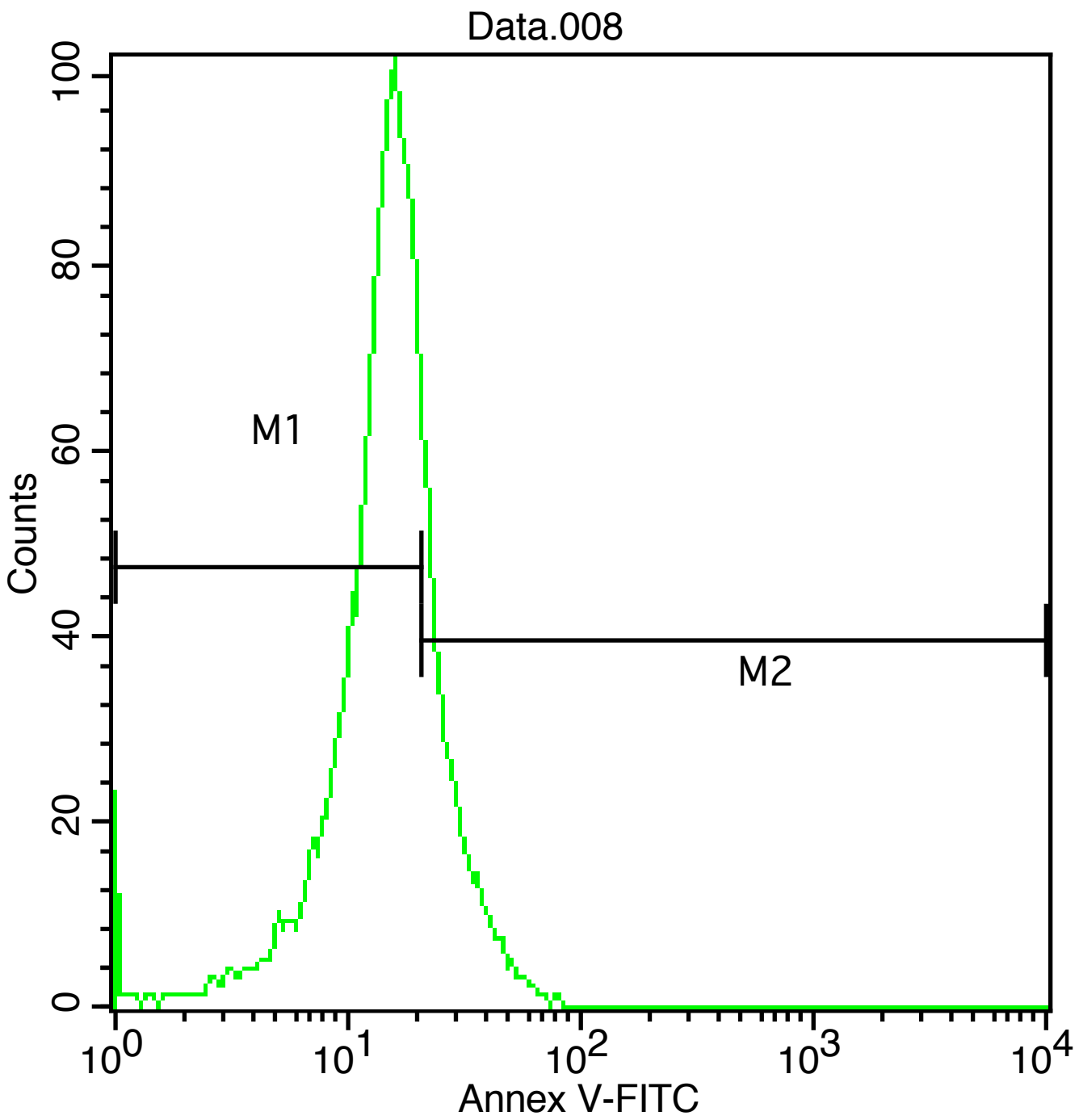

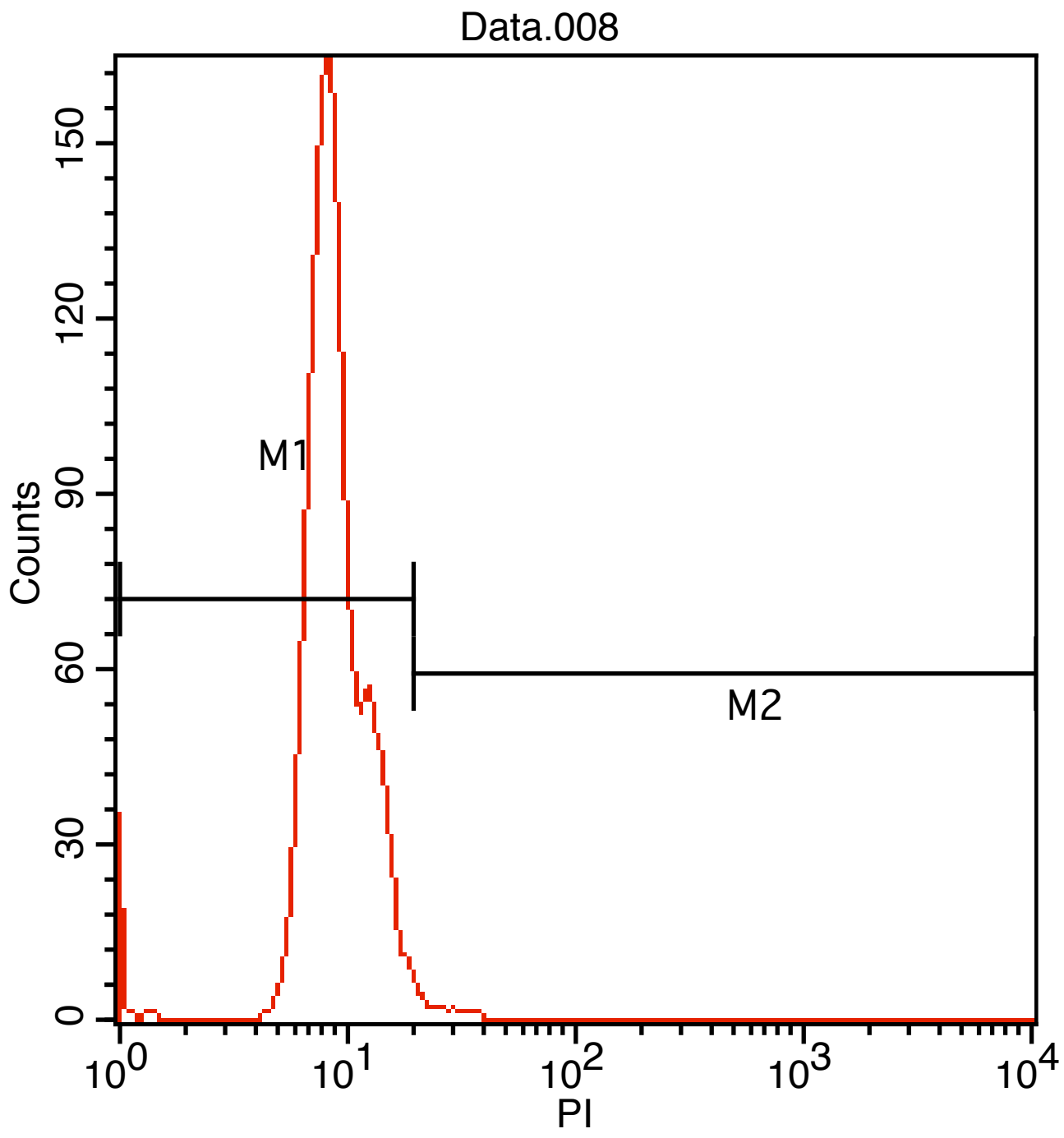

Histogram Statistics

File: Data.008
Sample ID: CaCo2 - 27
Tube: Untitled
Acquisition Date: 03-Mar-26
Gated Events: 9529
X Parameter: Annex V-FITC (Log)

Log Data Units: Linear Values
Patient ID: Apoptosis
Panel: Untitled Acquisition Tube List
Gate: G1
Total Events: 10000

Marker	Left, Right	Events	% Gated	% Total	Mean	Geo Mean	CV	Median	Peak Ch
All	1, 9910	9529	100.00	95.29	16.52	14.45	56.34	15.26	15
M1	1, 21	7544	79.17	75.44	13.23	12.17	33.04	13.82	15
M2	21, 9910	2030	21.30	20.30	28.85	27.57	41.59	25.48	21

Histogram Statistics

File: Data.008
Sample ID: CaCo2 - 27
Tube: Untitled
Acquisition Date: 03-Mar-26
Gated Events: 9529
X Parameter: PI (Log)

Log Data Units: Linear Values
Patient ID: Apoptosis
Panel: Untitled Acquisition Tube List
Gate: G1
Total Events: 10000

Marker	Left, Right	Events	% Gated	% Total	Mean	Geo Mean	CV	Median	Peak Ch
All	1, 9910	9529	100.00	95.29	9.21	8.68	37.56	8.35	8
M1	1, 19	9422	98.88	94.22	9.02	8.58	30.10	8.35	8
M2	19, 9910	109	1.14	1.09	26.20	24.98	41.94	23.50	20

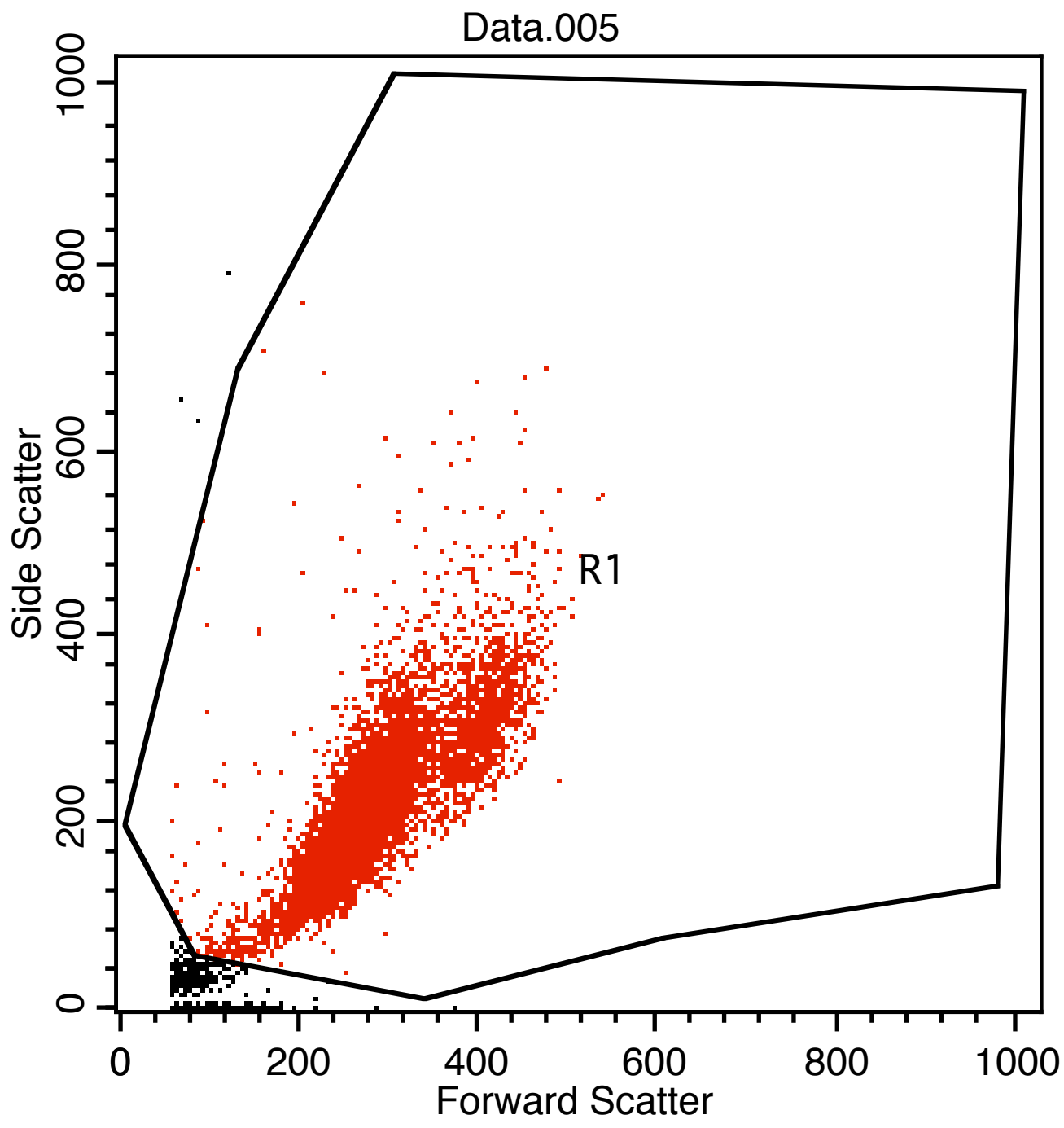

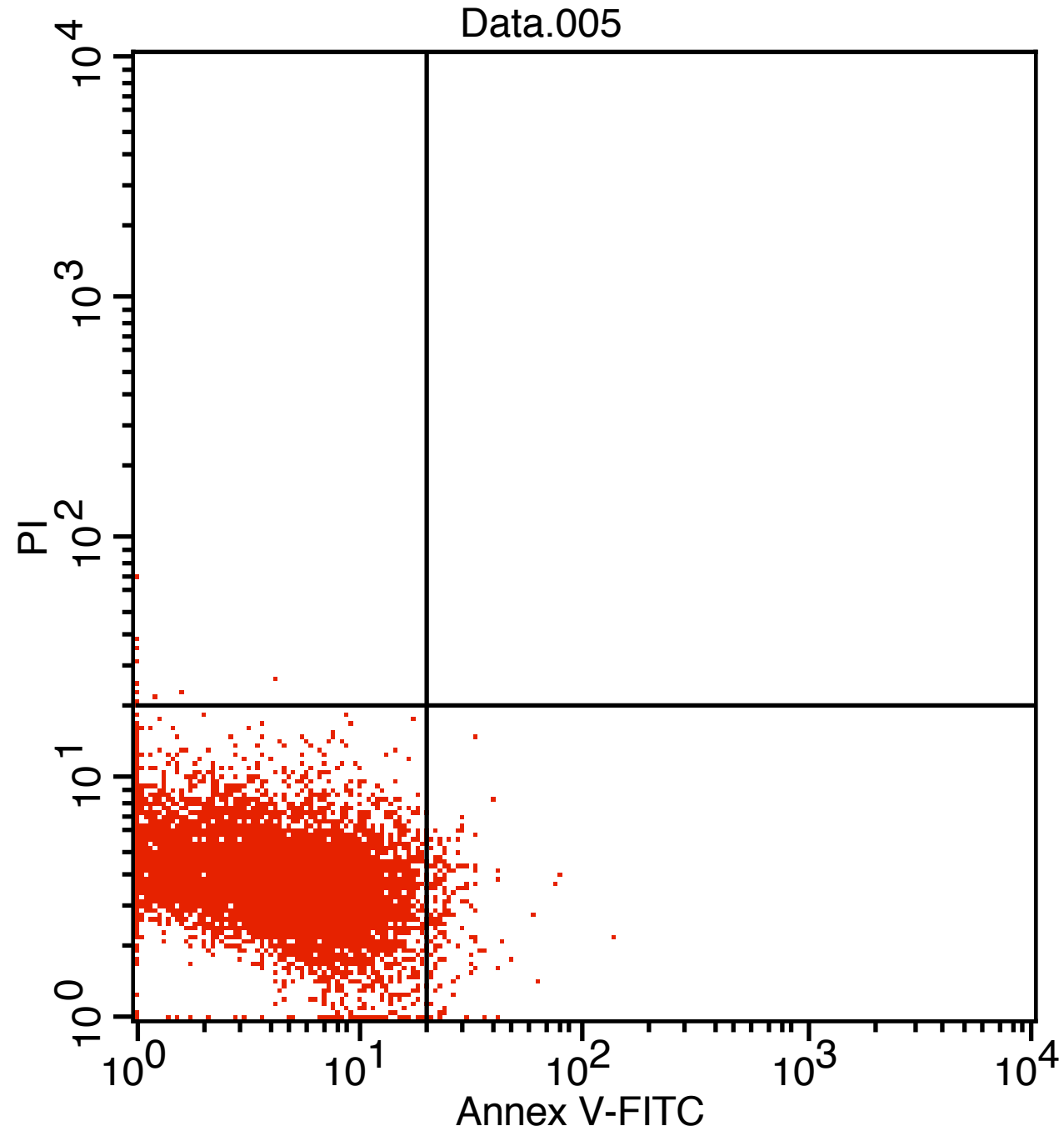

Quadrant Statistics

File: Data.005
Log Data Units: Linear Values
Sample ID: CaCo2 - Control (2)
Patient ID: Apoptosis
Tube: Untitled
Panel: Untitled Acquisition Tube List
Acquisition Date: 03-Mar-26
Gate: G1
Gated Events: 9510
Total Events: 10000
X Parameter: Annex V-FITC (Log)
Y Parameter: PI (Log)
Quad Location: 20, 20

Quad	Events	% Gated	% Total	X Mean	X Geo Mean	Y Mean	Y Geo Mean
UL	13	0.14	0.13	1.39	1.25	28.39	26.66
UR	0	0.00	0.00	***	***	***	***
LL	9344	98.25	93.44	4.86	3.49	4.26	3.87
LR	153	1.61	1.53	26.84	25.46	3.17	2.80

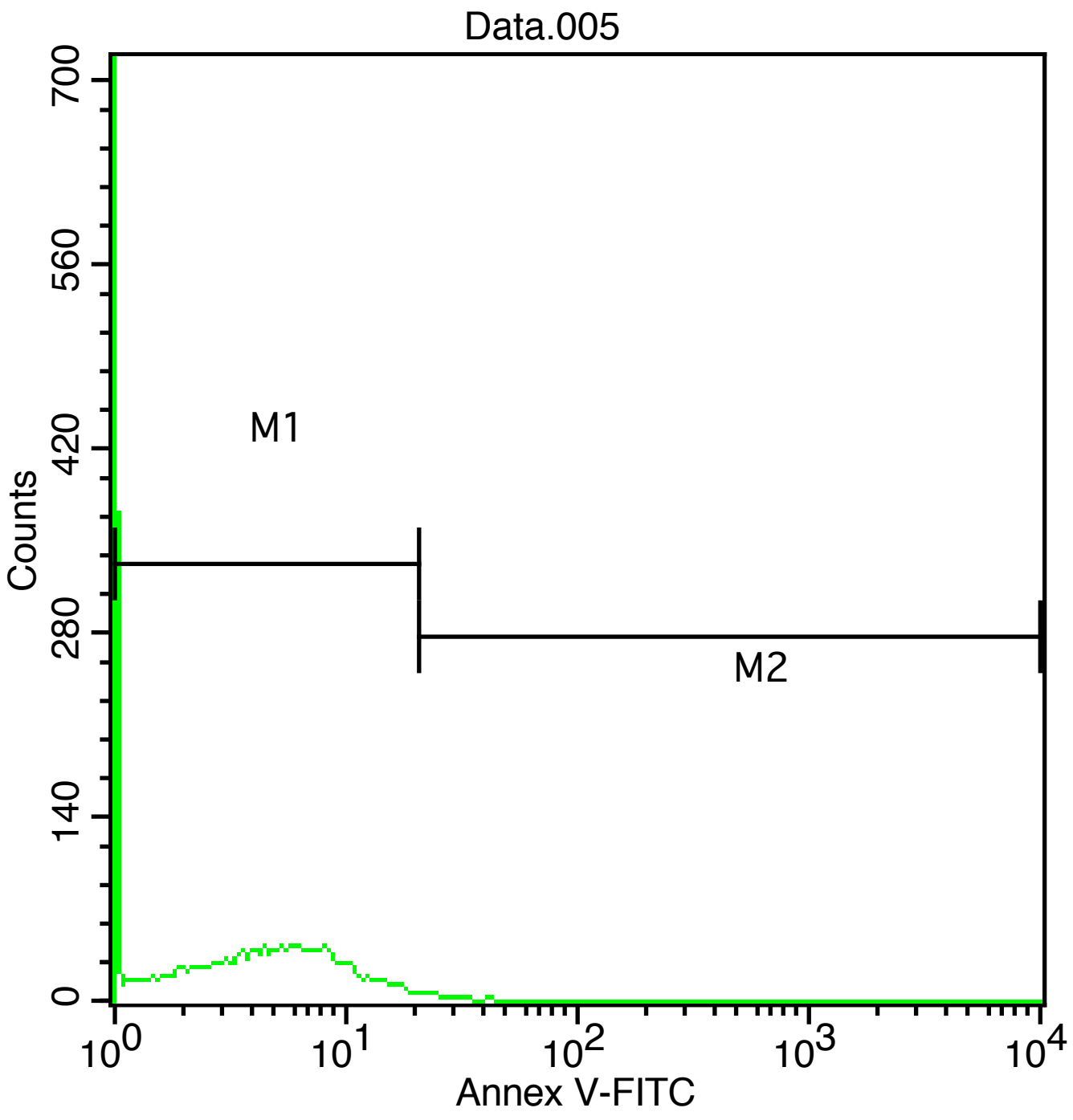

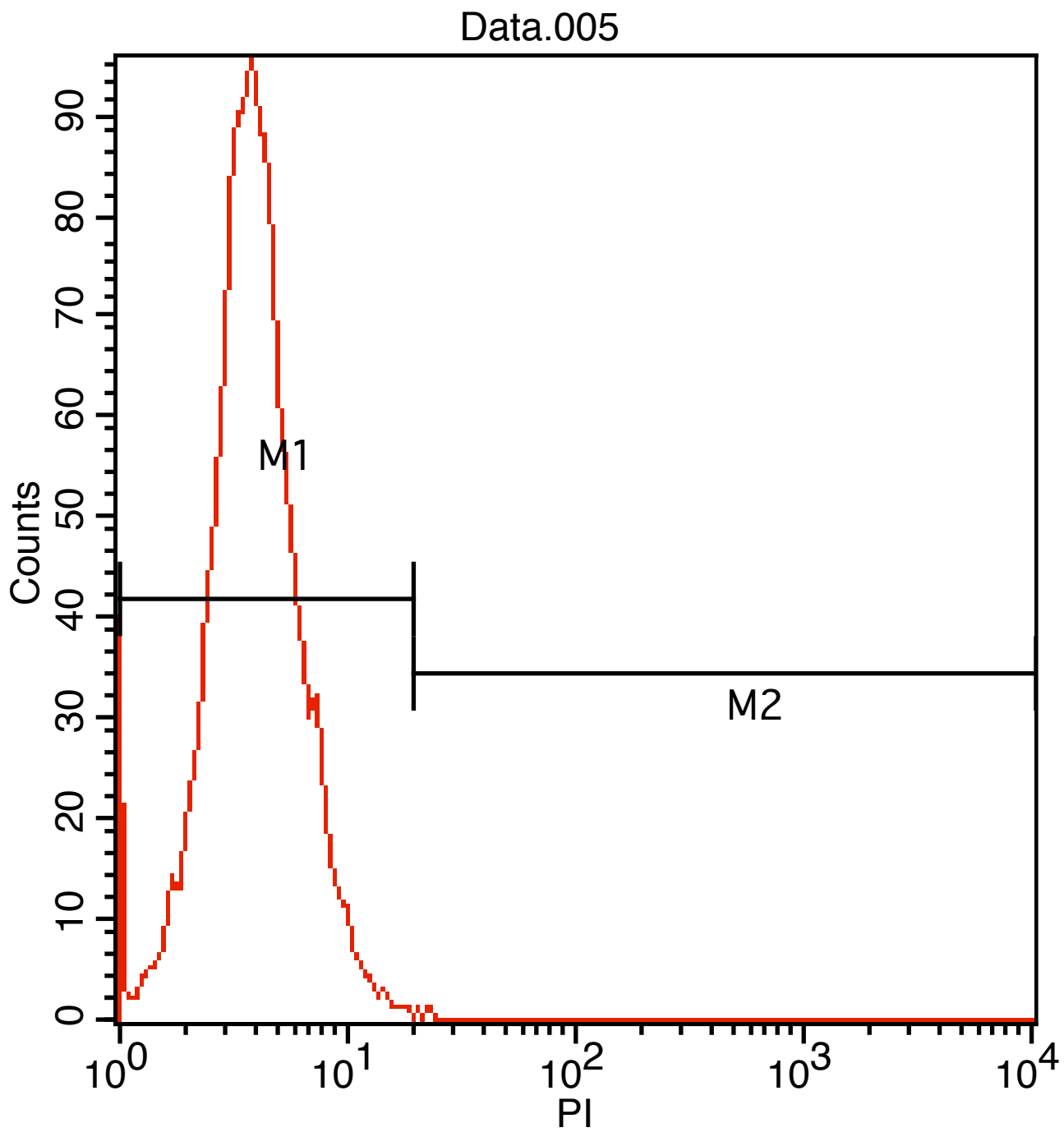

Histogram Statistics

File: Data.005
Sample ID: CaCo2 - Control (2)
Tube: Untitled
Acquisition Date: 03-Mar-26
Gated Events: 9510
X Parameter: Annex V-FITC (Log)

Log Data Units: Linear Values
Patient ID: Apoptosis
Panel: Untitled Acquisition Tube List
Gate: G1
Total Events: 10000

Marker	Left, Right	Events	% Gated	% Total	Mean	Geo Mean	CV	Median	Peak Ch
All	1, 9910	9510	100.00	95.10	5.21	3.60	95.37	3.92	1
M1	1, 21	9374	98.57	93.74	4.88	3.50	79.93	3.89	1
M2	21, 9910	139	1.46	1.39	27.53	26.09	46.12	23.50	20

Histogram Statistics

File: Data.005
Sample ID: CaCo2 - Control (2)
Tube: Untitled
Acquisition Date: 03-Mar-26
Gated Events: 9510
X Parameter: PI (Log)

Log Data Units: Linear Values
Patient ID: Apoptosis
Panel: Untitled Acquisition Tube List
Gate: G1
Total Events: 10000

Marker	Left, Right	Events	% Gated	% Total	Mean	Geo Mean	CV	Median	Peak Ch
All	1, 9910	9510	100.00	95.10	4.28	3.86	52.68	3.82	3
M1	1, 19	9496	99.85	94.96	4.24	3.85	47.48	3.80	3
M2	19, 9910	14	0.15	0.14	27.77	26.10	44.66	23.08	22

Molecular docking method

Molecular Operating Environment (MOE 2014) software was used to simulate the binding modes and calculate scores of the new candidates against HDAC-2 in comparison to those of the co-crystallized ligand. The three-dimensional structure of the selected proteins was downloaded from the PDB website. The water molecules and repeated chains were removed. Protons were added and the energy of the protein was minimized. The isolation of the pocket was then carried out. Validation of the downloaded structure was confirmed by re-docking the downloaded ligand into the isolated pocket. The obtained root mean square deviation (RMSD) was found to be lower than 1.5 Å. The preparation of the new antitumor derivatives for docking was carried out by the construction of the chemical structures at MOE. Protons were then added to the 3D structure. Finally, the energy was minimized using Force Field MMFF94x. The prepared structures were added to the created database. MOE conducted the docking of the newly synthesized compounds, calculated the binding energies, and provided the binding modes of all of them. Discovery studio 4 software was used to visualize the binding patterns.

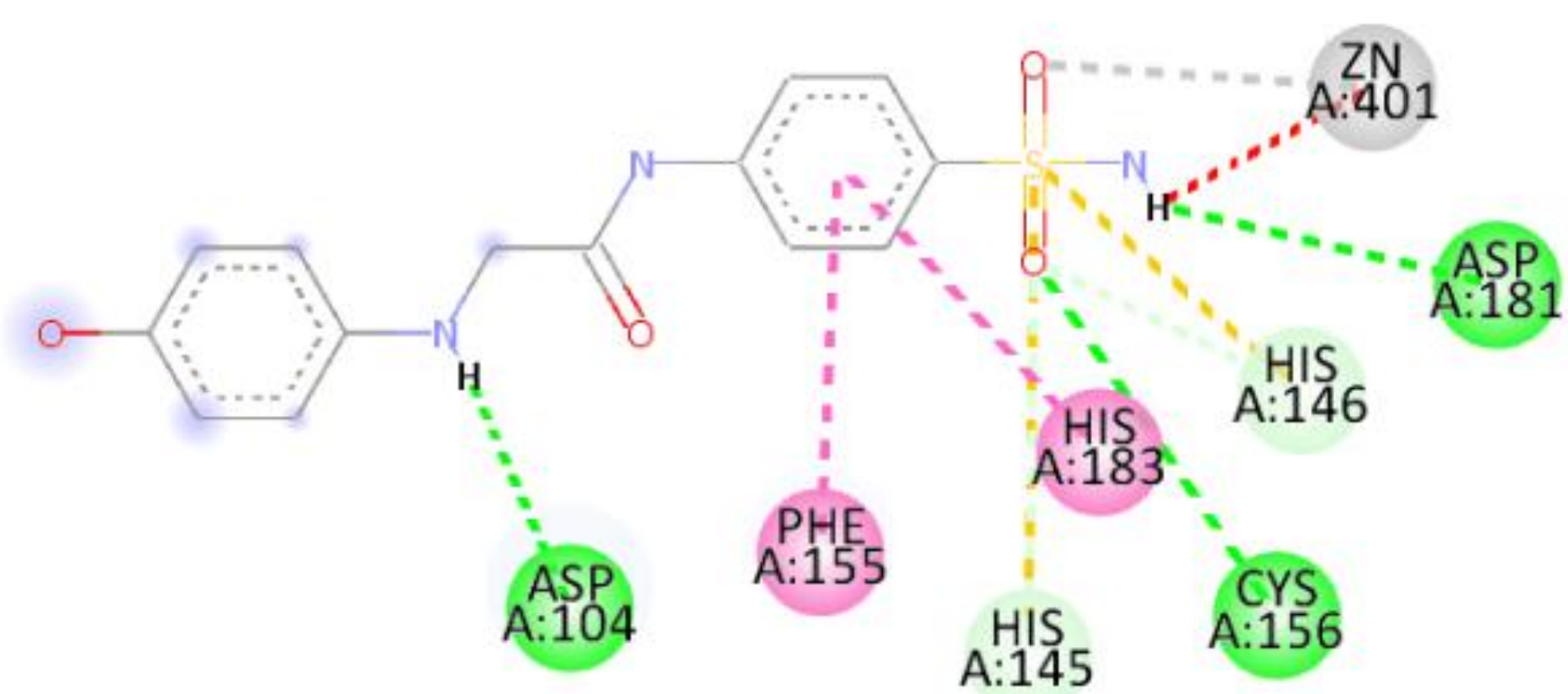

Fig. S1: 2D image for binding mode of compound 9

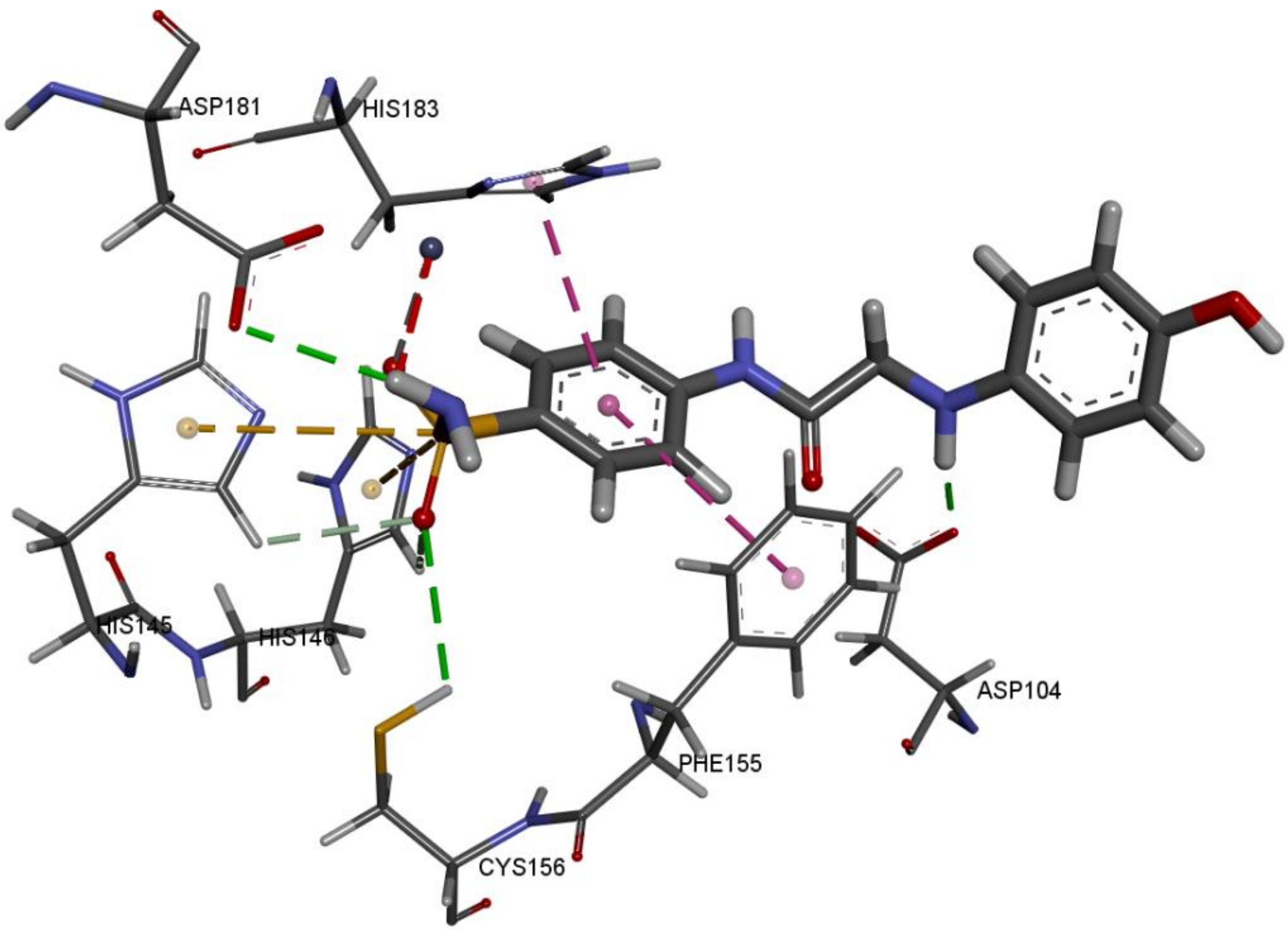

Fig. S2: 3D illustration for binding mode of compound 9

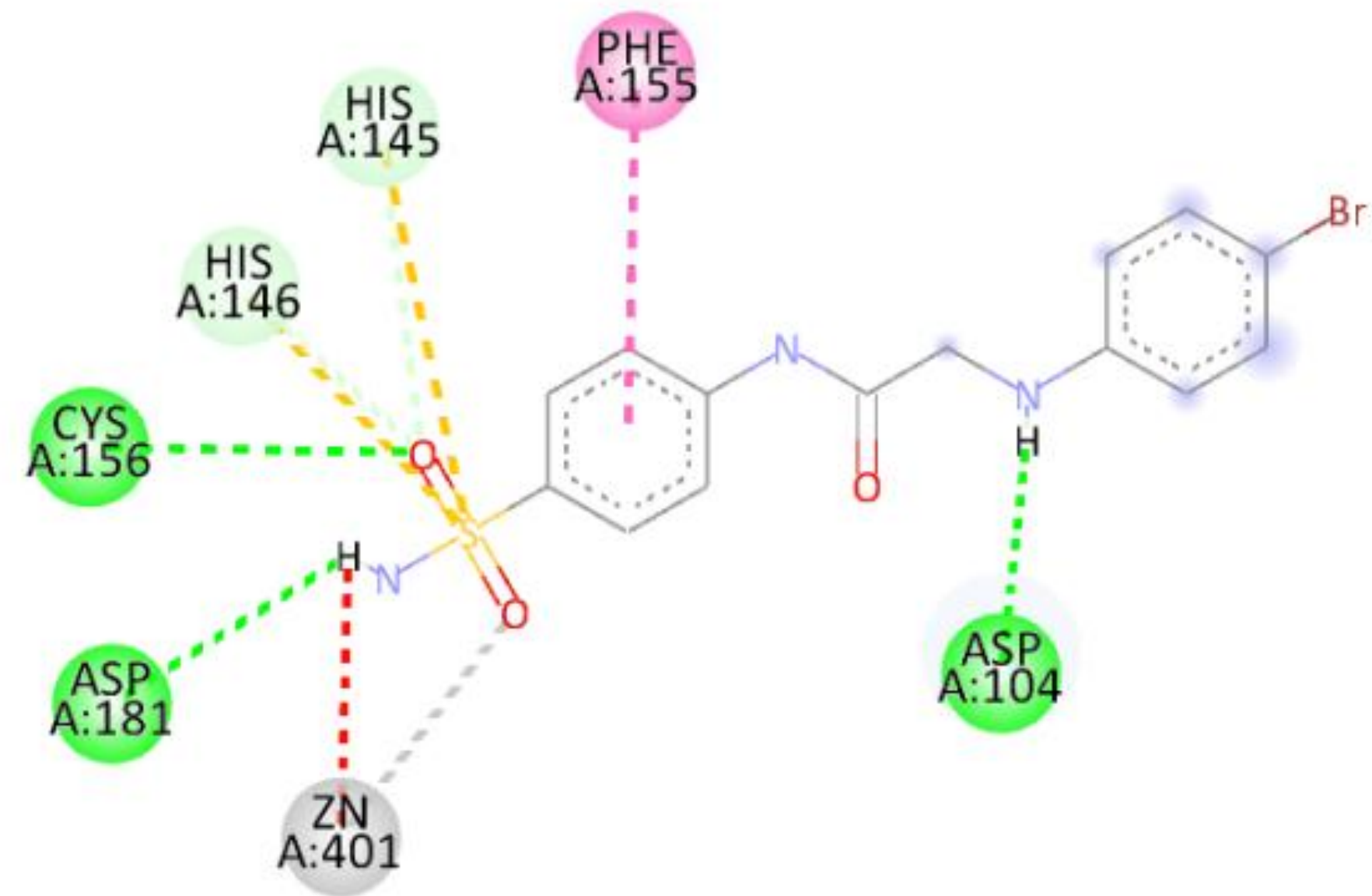

Fig. S3: 2D image for binding pattern of compounds 11

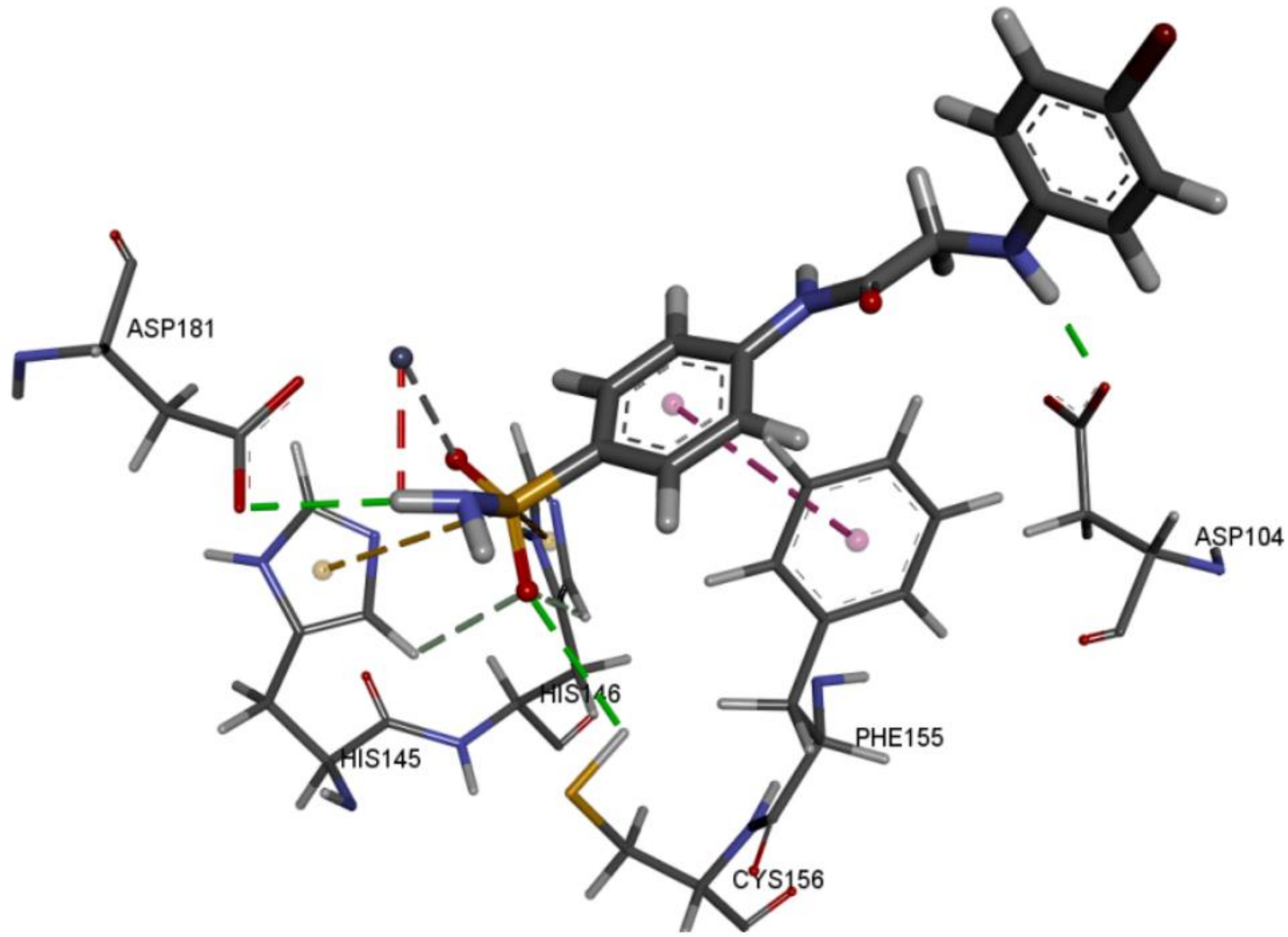

Fig. S4: 3D illustration for binding pattern of compound 11
